# Timely Updating on Ber/Geo/1/2 Queue Modeled Status Updating System with Eavesdropper

**DOI:** 10.3390/e27090972

**Published:** 2025-09-18

**Authors:** Jixiang Zhang, Han Xu, Anqi Zheng, Daming Cao, Yinfei Xu, Chengyu Lin

**Affiliations:** 1 School of IoT Engineering, Wuxi Taihu University, Wuxi 214063, China; zhangjx@wxu.edu.cn (J.Z.); zhengaq@wxu.edu.cn (A.Z.); 2 Provincial Key Laboratory of Intelligent Internet of Things Technology and Applications, Wuxi Taihu University, Wuxi 214063, China; 3 School of Information Science and Engineering, Southeast University, Nanjing 210096, China; han_xu@seu.edu.cn (H.X.); yinfeixu@seu.edu.cn (Y.X.); 4 School of Electrical and Information Engineering, Nanjing University of Information Science and Technology, Nanjing 210044, China; dmcao@nuist.edu.cn

**Keywords:** age of information, probabilistic deletion, information freshness, transmission security, wiretap channel

## Abstract

We consider that the source sends packets to the receiver through a Ber/Geo/1/2 queue modeled status updating system, where the transmitted packets are subject to potential eavesdropping. Time is discretized into identical time slots. This paper studies the tradeoffs between the information freshness and transmission security of a system, where freshness is characterized by the age of information (AoI) metric and transmission security is represented by the proportion of obtained insecure packets over a long period of time. We assume that in a time slot, the source generates a new packet with probability *p*, and a packet arrives at the receiver with probability $\gamma_{d}$. With probability $\gamma_{E}$, a transmitted packet is eavesdropped. At the receiver, AoI is defined as the elapsed time since the generation instant of the latest obtained packet. A packet is defined to be insecure if it is obtained by the eavesdropper earlier than the receiver. To control the proportion of insecure packets obtained in the receiver, we propose using the probabilistic deletion/retaining scheme. More specifically, when a packet is eavesdropped before arriving at the receiver, this packet is deleted with probability δ or retained with probability 1−δ. Under this transmission policy, we derive the system’s average AoI which we call the average δ−secure AoI, and investigate its relations with the insecure packet proportion, which is denoted as η(δ). The obtained formulas are then calculated in three special cases, including $\gamma_{E}=0$, $\gamma_{E}=1$, and δ=1. We explain that these cases correspond to the average AoI of a basic status updating system with Ber/Geo/1/2 queue, packet with random geometric deadline in service process, and average age of secure information (AoSI), respectively. Numerical simulations of obtained results are provided. In particular, the tradeoffs between average δ−secure AoI and η(δ) are analyzed in detail. We demonstrate that depending on the value of the eavesdropping probability $\gamma_{E}$, average δ−secure AoI varies in different trends with η(δ), and in most cases the average δ−secure AoI and η(δ) can be minimized simultaneously.

## 1. Introduction

Due to the rapid development of wireless communication networks, in recent years IoT technology has given rise to a large number of emerging applications. Among them, real-time data transmission plays an important role. One typical application is autonomous vehicles. In order to achieve intelligent and safe autonomous driving, different kinds of sensors installed on vehicles need to provide real-time feedback about the surrounding road conditions to the vehicle’s control center so that the speed, steering, and other driving states can be updated in a timely manner. In papers [1,2], Kaul and Yates et al. proposed using age of information (AoI) to measure the freshness of packets when they arrive at the receiver. Then, the freshness of the obtained packets represents the transmission timeliness performance of the system. Since then, as a metric characterizing packet freshness, AoI has been analyzed theoretically and been widely used in system design or scheduling problems.

Based on queuing theory, Yates et al. proposed a status updating system as a real-time communication model. They defined the age of information as the random process of current time minus u(t), where u(t) is the instant that the latest obtained packet was generated. The average AoI of the system with basic queue was obtained in [1,2,3,4,5]. In particular, in work [5], the authors proved that a small-capacity system has a lower average AoI and preempting/replacingold packet helps to reduce the average AoI of the system. On the M/M/1/2 queue modeled system, in paper [6] Kam et al. demonstrated that by controlling the packet waiting time in the buffer, imposing an appropriate packet deadline can achieve a lower average AoI. Inoue proved the same conclusion for an infinite capacity system in work [7]. In another paper [8], by deriving a general formula which links the stationary distributions of AoI, peak AoI, and service time, Inoue determined most results about AoI on single-source single-server systems. Assuming that there are multiple sources, the AoI of every source was analyzed for example in works [9,10,11,12], while a system with multiple servers was investigated in papers [13,14,15]. Notice that analyzing AoI for a multiple-server system is more complex because sometimes the receiver may obtain one packet that is even older than that which the receiver currently has. In these cases, AoI will not be updated. In addition, AoI has often been used as timeliness measure, sometimes even as an optimization objective in designing optimal resource scheduling problems, such as in [16,17,18,19,20,21,22]. In recent years, many results have been obtained in deriving the average AoI and the stationary distribution of AoI for discrete time status updating systems. In [23,24,25,26], the authors determined the average AoI of system with several simple queuing models, such as FCFS Ber/G/1/1, Ber/G/1/∞, and a system with multiple sources. In previous works [27,28,29], the authors derived the average value and stationary distribution of discrete time AoI for Ber/Geo/1/1, Ber/Geo/1/2, and Ber/Geo/1/∞ queue modeled systems. In addition, the discrete age of information for systems with multiple prioritized sources was discussed in paper [30].

When transmitting packets via electromagnetic waves in open spaces, an illegal eavesdropper may eavesdrop on the transmitted packets through a wiretap channel. Recently, it was found that within the framework of AoI theory, constructing some new security measures based on real-time transmission performance, then studying the tradeoffs between transmission timeliness and transmission security of the system has become a noticeable research topic. Unlike the usual secure transmission research such as in works [31,32,33,34], in real-time communication scenario with eavesdroppers, one packet is defined to be insecure if it is obtained by the eavesdropper before arriving at the receiver.

In papers [35,36], using the short-packet permutation-based policy, Yang considered transmitting packets securely and in a timely manner, where an eavesdropper is present to probe their communication link. They proposed a new security measure called secrecy margin, which was defined as the area of random region enclosed by the AoI sample path from $t_{B}$ to $t_{E}$, where $t_{B}$ is the instance that the receiver obtains a packet, and $t_{E}$ denotes the instance that the same packet is eavesdropped. By maximizing the average secrecy margin, they determined the optimal structure of short packets. Later, the same real-time and secure communication scenario was studied by Wang and Chen et al. in work [37]. To characterize transmission security, they constructed another two AoI-based security measurements by drawing inspiration from the concept of Wyner’s secrecy capacity [38]. They defined secrecy age to be average instantaneous gap between the eavesdropper’s and receiver’s AoI, and the secrecy age outage probability denoted the probability that this gap does not exceed some threshold. Under random and threshold-based packet generation policies, the optimal generation probability/packet transmission strategy was obtained by maximizing the joint measure, i.e., average secrecy age divided by average secrecy age outage probability. Age of secure information (AoSI) was defined and investigated in [39,40]. Just as its name implies, AoSI characterizes the evolution of the real-time age of the latest and secure packet in the receiver, and average AoSI equals average AoI calculated over those secure packets. In [39], the authors analyzed AoSI using finite block-length information theory, while [40] considered the situation where the eavesdropper is an energy harvesting node. The optimal transmission strategy that minimizes average AoSI was obtained by the certain optimization method. In work [41], considering the scenarios where packets are transmitted through infinite capacity systems, we defined the freshness advantage *F* to be the security measurement. Then, the relations and tradeoffs between transmission timeliness and transmission security were investigated. We proved that the average AoI of legitimate receivers and the freshness advantage cannot be minimized simultaneously. Therefore, there exist certain tradeoffs between reducing the average AoI and maintaining a sufficiently large freshness advantage *F*.

Assuming that there is an eavesdropper, this paper considers transmitting packets over Ber/Geo/1/2 queue modeled status updating systems, and analyzes the relations between packet freshness and transmission security, where freshness is characterized using average AoI while security performance is measured by the proportion of obtained insecure packets. The motivation of considering systems with Ber/Geo/1/2 queue are explained as follows. Firstly, notice that the system with capacity 1 buffer is the simplest buffer-equipped transmission system, and this model simplifies the analysis and calculations for both average AoI and insecure packet proportion. In addition, although the numerical simulations in work [5,6] demonstrated that the average AoI of the M/M/1/2 queue modeled system almost has no advantage over that of the system with the M/M/1/1 queue, reserving some arriving packets waiting for transmission not only improves utilization of the source node’s energy but the packets themselves may convey some useful information, such as synchronization signals and the estimation state of wireless channels.

In order to control the number of insecure packets arriving at the receiver, we adopt a probabilistic deletion/retaining transmission scheme. Specifically speaking, in each time slot, an insecure packet is deleted with probability δ, and with probability 1−δ this packet is retained and transmission continues. Here, we explain that deleting one insecure packet may be very effective. In real-time communication scenarios, in order to reduce the average AoI, usually each packet has a few bytes such that transmitting one packet takes a short time. Consequently, it is necessary not for an eavesdropper to have a strong eavesdropping ability for a long time but for the eavesdropper to have a strong eavesdropping ability for a short period of time. Then, with large probability one packet can be eavesdropped. Considering that each packet contains only a small amount of data, we believe that in real-time applications, such as autonomous vehicles, deleting an insecure packet directly is more appropriate than using some secure transmission policies, for example, encrypting the transmitted packets.

Based on system models and transmission policy, we derive the average AoI of the system, which we call average δ−secure AoI, ${\bar{\Delta }}_{\delta }$. In addition, as the function of deletion probability δ, the proportion of insecure packets obtained in the receiver, η(δ), is also determined. Combining ${\bar{\Delta }}_{\delta }$ and η(δ), the relationships between transmission timeliness and transmission security can be analyzed. Provided ${\bar{\Delta }}_{\delta }$ and η(δ), actually we establish the following achievability relationships: we can achieve average AoI ${\bar{\Delta }}_{\delta }$ under the restriction that the proportion of the obtained insecure packet is at most η(δ).

In this paper, we use insecure packet proportion η(δ) to be the transmission security measurement. Here, we propose one situation where the insecure packets proportion can be linked to the amount of information leaked to the eavesdropper. Consider that the packets are encoded to be identical-length codewords, for example, by maximum distance separable (MDS) codes. Assuming that each codeword is composed of *L* symbols, and decoding a codeword requires at least K<L symbols, in the legitimate receiver, as long as η(δ) is controlled below K/L, with large probability the eavesdropper cannot restore the original packet, and thus obtains no information. By introducing encoding and decoding procedures, one can establish the relationships between η(δ) and average leaked information. In practical applications such as autonomous driving, encoding packets are usually necessary, which facilitates the unified processing of packets in the entire system. Under the redundancy protection mechanism brought by encoding strategies, allowing some packets to be insecure rather than waiting for enough secure packets can advance the decoding time at the receiver, thereby accelerating the response speed of autonomous cars. Using packet encoding and decoding provides helpful inspiration for the design of real-time systems like autonomous vehicles and some others.

The main contributions of this paper are summarized as follows.

1. Under probabilistic deletion/retaining transmission policy, we derive the formula of average δ−secure AoI and the insecure packet proportion η(δ). To verify the correctness of the average AoI formula, we prove that in cases of $\gamma_{E}=0$, average δ−secure AoI reduces to ${\bar{\Delta }}_{Ber/Geo/1/2}$, i.e., the average AoI of system with basic Ber/Geo/1/2 queues.
2. We further consider two special cases. In the first one, $\gamma_{E}=1$, and in the second case, δ=1. We determine the average AoI for both cases and explain that when $\gamma_{E}=1$, the considered problem degenerates to analyzing AoI for the system with a random service time deadline, while by setting δ=1, the average δ−secure AoI is actually equal to the average AoSI of the system.
3. The relationships between ${\bar{\Delta }}_{\delta }$ and η(δ) are investigated through numerical simulations. We demonstrate that depending on the value of $\gamma_{E}$, the average δ−secure AoI ${\bar{\Delta }}_{\delta }$ varies in different trends with η(δ). In most cases, especially when $\gamma_{d}>\gamma_{E}$, it is shown that ${\bar{\Delta }}_{\delta }$ and η(δ) can be minimized simultaneously by setting δ=1.
4. For a system with random service time deadline, numerical simulations show that in most cases, imposing such a deadline cannot reduce the average AoI. This result actually negates the effectiveness of imposing a service time deadline in improving transmission timeliness, at least in Ber/Geo/1/2 queue modeled systems.
5. In the Discussion and Conclusion section, we provide brief comments discussing the rationality of different security measures proposed in real-time communication scenarios.

The rest of the paper is organized as follows. We introduce the system model, the parameters, and transmission policy in Section 2. By constructing a four-dimensional state vector and calculating the probability generation function of AoI, we derive the formula of average δ−secure AoI in Section 3. It is verified that when $\gamma_{E}=0$, average δ−secure AoI degenerates to ${\bar{\Delta }}_{Ber/Geo/1/2}$, which checks the correctness of the obtained formula. In Section 4, two other special cases are analyzed, where we let $\gamma_{E}=1$ in the first one and δ=1 in the second one. The average AoI for both cases are determined. In Section 5, we provide numerical simulation for the results obtained in Section 3 and Section 4. The relationships between ${\bar{\Delta }}_{\delta }$ and η(δ) are investigated by analyzing numerical examples. In particular, we show that when changing the value of $\gamma_{E}$, ${\bar{\Delta }}_{\delta }$ varies in different trends with η(δ). Finally, in Section 6, we conclude the paper, make brief comments on the security measures, and discuss some possible further works.

## 2. System Model and Problem Formulation

**Figure 1:**
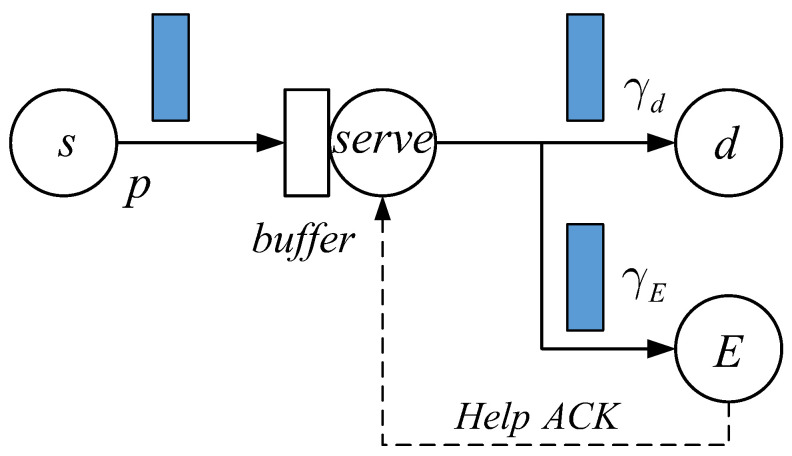
Ber/Geo/1/2 queue modeled status updating system with probabilistic packet deletion during the service process incurred by eavesdropping. Packet arrivals form the Bernoulli process. The objective is studying the relationships between the transmission timeliness and transmission security, which are represented by the average AoI of the legitimate receiver and the proportion of insecure packets that the receiver obtains over a long period of time.

We describe the considered status updating system depicted in Figure 1.

At each time slot, the source *s* generates a new packet with probability *p*. Following the First Come-First Served (FCFS) rule, these packets are delivered to the receiver *d* through the transmitter. The newly arrived packets that are unable to be served are temporarily stored in the capacity 1 buffer. When there are packets in both the server and the buffer, other arriving new packets are blocked and discarded. At the receiver, the age of information (AoI) is defined as the difference between the current time and the generation instant of the latest obtained packet. The value of AoI increases by 1 after each time slot if no newer packet is obtained. Every time a fresher packet, with a smaller age, arrives at *d*, this packet replaces the old one, and the value of the AoI is reduced to its instantaneous age. Long-term average AoI or other AoI-related quantities have been used widely to measure the freshness of obtained packets and demonstrate the system’s timeliness of transmitting status updates. Obviously, a lower average AoI means the system has better real-time transmission performance.

During packet transmissions from the source to the receiver, it is assumed that an eavesdropper attempts to probe the communication link between *s* and *d* and steal the information contained in the packets. Due to signal fadings and the unreliability of wireless channels, in the considered model, we assume that a transmitted packet is obtained at the legitimate receiver with probability $\gamma_{d}$, and the successful eavesdropping probability is equal to $\gamma_{E}$. Equivalently, both transmission links are modeled as i.i.d. erasure channels. In general situations, we have $\gamma_{d}>\gamma_{E}$. That is, for each packet the probability of successfully transmitting to a legitimate receiver is higher than that of being eavesdropped. If a transmitted packet is eavesdropped before arriving at *d*, we define it as an insecure packet. Once a packet becomes insecure due to eavesdropping, we assume that a help ACK signal is sent back to the transmitter. Therefore, the transmitter knows whether the current packet is secure or not at all times.

The goal of this paper is deriving achievable average AoI under the condition that the proportion of insecure packets that the receiver obtains is controlled. To do this, we apply the random deletion/retaining transmission scheme. More specifically, we consider that at the beginning of each time slot, with probability δ, the transmitter discards an insecure packet and starts sending the next new and secure one, if there is such a packet, in the buffer. Otherwise, with probability 1−δ, the transmitter will still send the insecure packet in this time slot. If this packet is not obtained by the receiver, this probabilistic deletion/retaining scheme is performed again at the beginning of the next time slot until this insecure packet arrives at *d*, or is deleted by the transmitter. Based on the proposed system assumptions and the above transmission scheme, in this paper we derive the average AoI of the system, which we call the average δ−secure AoI. Provided average δ−secure AoI and insecure packet proportion, the relationships between packet freshness and transmission security are investigated in depth.

## 3. Analysis of Age of δ−Secure Information for Ber/Geo/1/2 Queue Modeled Status Updating System

As a whole, we define four-dimensional state vectors (n,m,l,0) and (n,m,l,1) to represent the AoI, age of packet under the service, and age of packet in the buffer. The last component 0 indicates that the currently served packet is secure, while 1 denotes that the packet in the server is insecure. Here, there is nothing special about 0 and 1; they can be replaced by any two distinguishable notations. In the following, we show that the random transfers of (n,m,l,0) and (n,m,l,1) can be described clearly, and average δ−secure AoI is obtained by analyzing the steady state of the four-dimensional random process constructed by (n,m,l,0) and (n,m,l,1).

In a time slot, we define binary random variables *A*, $B_{d}$, $B_{E}$, and *F* to represent if the source generates a new packet, if the transmitted packet arrives at the receiver and the eavesdropper, and whether the transmitter deletes the insecure packet. According to different values of these variables, we list all of the state vector transfers in Table 1. State vectors (n,m,0,0), (n,m,0,1) denote that the buffer is empty, and the packet under service is secure if the last component is 0, or is insecure when the last component is 1. The last case (n,0,0,0) means that both the server and the buffer are empty. Since there is no packet in the server, in these cases, the last component 0 does not indicate a secure packet.

Brief explanations are given for state vector transfers in Table 1. For initial state vector (n,m,l,0), a secure packet of age *m* is under the service. The realizations of $B_{d}$ and $B_{E}$ represent the packet being transmitted to *d* and *E*. For the case of $B_{d}=0,B_{E}=0$, it means that at (the right endpoint of) this time slot, neither the receiver nor the eavesdropper obtains the packet, and thus the state vector changes to (n+1,m+1,l+1,0). If $B_{d}=0,B_{E}=1$, that is, the eavesdropper obtains the packet but the receiver does not, in this case, the transmitted packet becomes insecure and the random deletion/retaining scheme is performed. The insecure packet is deleted with probability δ, which is denoted by F=1, resulting in the state vector transferring to (n+1,l+1,0,0). Since the new packet of age l+1 is secure, the last component is reset to 0. On the contrary, with probability 1−δ, i.e., when F=0, the insecure packet is retained, and it sees that the initial state vector (n,m,l,0) changes to (n+1,m+1,l+1,1). According to similar discussions, it is easy to determine the state vector transfers under the conditions $B_{d}=1,B_{E}=0$ and $B_{d}=1,B_{E}=1$, and that from other initial states. It is worth noticing that for (n,m,l,1) and (n,m,0,1), the packet under the service is insecure because it has been obtained by an eavesdropper. Therefore, when describing state vector transfers, we only need to consider the realizations of *A*, $B_{d}$, and *F*.

Assuming that the constructed random process reaches the steady state, according to the probability balance conditions, we determine the stationary equations as follows:

(1) $$\left\{\begin{array}{c} \pi_{\left(n,m,l,0\right)}=\pi_{\left(n-1,m-1,l-1,0\right)}\left(1-\gamma_{d}\right)\left(1-\gamma_{E}\right)\;\;\;\;\;\;\;\;\;\;\left(n>m>l\geq 2\right)\\ \pi_{\left(n,m,1,0\right)}=\pi_{\left(n-1,m-1,0,0\right)}p\left(1-\gamma_{d}\right)\left(1-\gamma_{E}\right)\;\;\;\;\;\;\;\;\;\;\;\;\left(n>m\geq 2\right)\\ \pi_{\left(n,m,l,1\right)}=\pi_{\left(n-1,m-1,l-1,0\right)}\left(1-\gamma_{d}\right)\gamma_{E}\left(1-\delta \right)+\pi_{\left(n-1,m-1,l-1,1\right)}\left(1-\gamma_{d}\right)\left(1-\delta \right)\;\;\;\left(n>m>l\geq 2\right)\\ \pi_{\left(n,m,1,1\right)}=\pi_{\left(n-1,m-1,0,0\right)}p\left(1-\gamma_{d}\right)\gamma_{E}\left(1-\delta \right)+\pi_{\left(n-1,m-1,0,1\right)}p\left(1-\gamma_{d}\right)\left(1-\delta \right)\;\;\;\;\;\;\left(n>m\geq 2\right)\\ \pi_{\left(n,m,0,0\right)}=\pi_{\left(n-1,m-1,0,0\right)}\left(1-p\right)\left(1-\gamma_{d}\right)\left(1-\gamma_{E}\right)+\sum_{k=n}^{\infty }\pi_{\left(k,n-1,m-1,0\right)}\gamma_{d}\left(1-\gamma_{E}\delta \right)+\sum_{j=m}^{n-2}\pi_{\left(n-1,j,m-1,0\right)}\gamma_{E}\delta \\ \;\;\;+\sum_{k=n}^{\infty }\pi_{\left(k,n-1,m-1,1\right)}\gamma_{d}\left(1-\delta \right)+\sum_{j=m}^{n-2}\pi_{\left(n-1,j,m-1,1\right)}\delta \;\;\;\;\;\;\left(n>m\geq 2,n-m\geq 2\right)\\ \pi_{\left(m+1,m,0,0\right)}=\pi_{\left(m,m-1,0,0\right)}\left(1-p\right)\left(1-\gamma_{d}\right)\left(1-\gamma_{E}\right)+\sum_{k=m+1}^{\infty }\pi_{\left(k,m,m-1,0\right)}\gamma_{d}\left(1-\gamma_{E}\delta \right)\\ \;\;\;+\sum_{k=m+1}^{\infty }\pi_{\left(k,m,m-1,1\right)}\gamma_{d}\left(1-\delta \right)\;\;\;\;\;\;\;\;\;\;\;\;\left(m\geq 2\right)\\ \pi_{\left(n,1,0,0\right)}=\pi_{\left(n-1,0,0,0\right)}p\left(1-\gamma_{d}\right)\left(1-\gamma_{E}\right)+\sum_{k=n}^{\infty }\pi_{\left(k,n-1,0,0\right)}p\gamma_{d}\left(1-\gamma_{E}\delta \right)+\sum_{j=1}^{n-2}\pi_{\left(n-1,j,0,0\right)}p\gamma_{E}\delta \\ \;\;\;+\sum_{k=n}^{\infty }\pi_{\left(k,n-1,0,1\right)}p\gamma_{d}\left(1-\delta \right)+\sum_{j=1}^{n-2}\pi_{\left(n-1,j,0,1\right)}p\delta \;\;\;\;\;\;\;\left(n\geq 3\right)\\ \pi_{\left(2,1,0,0\right)}=\pi_{\left(1,0,0,0\right)}p\left(1-\gamma_{d}\right)\left(1-\gamma_{E}\right)+\sum_{k=2}^{\infty }\pi_{\left(k,1,0,0\right)}p\gamma_{d}\left(1-\gamma_{E}\delta \right)+\sum_{k=2}^{\infty }\pi_{\left(k,1,0,1\right)}p\gamma_{d}\left(1-\delta \right)\\ \pi_{\left(n,m,0,1\right)}=\pi_{\left(n-1,m-1,0,0\right)}\left(1-p\right)\left(1-\gamma_{d}\right)\gamma_{E}\left(1-\delta \right)+\pi_{\left(n-1,m-1,0,1\right)}\left(1-p\right)\left(1-\gamma_{d}\right)\left(1-\delta \right)\;\left(n>m\geq 2\right)\\ \pi_{\left(n,1,0,1\right)}=\pi_{\left(n-1,0,0,0\right)}p\left(1-\gamma_{d}\right)\gamma_{E}\left(1-\delta \right)\;\;\;\;\;\;\;\;\;\;\;\left(n\geq 2\right)\\ \pi_{\left(n,0,0,0\right)}=\pi_{\left(n-1,0,0,0\right)}\left[\left(1-p\right)+p\gamma_{E}\delta \right]+\sum_{k=n}^{\infty }\pi_{\left(k,n-1,0,0\right)}\left(1-p\right)\gamma_{d}\left(1-\gamma_{E}\delta \right)+\sum_{j=1}^{n-2}\pi_{\left(n-1,j,0,0\right)}\left(1-p\right)\gamma_{E}\delta \\ \;\;\;+\sum_{k=n}^{\infty }\pi_{\left(k,n-1,0,1\right)}\left(1-p\right)\gamma_{d}\left(1-\delta \right)+\sum_{j=1}^{n-2}\pi_{\left(n-1,j,0,1\right)}\left(1-p\right)\delta \;\;\;\;\;\left(n\geq 3\right)\\ \pi_{\left(2,0,0,0\right)}=\pi_{\left(1,0,0,0\right)}\left[\left(1-p\right)+p\gamma_{E}\delta \right]+\sum_{k=2}^{\infty }\pi_{\left(k,1,0,0\right)}\left(1-p\right)\gamma_{d}\left(1-\gamma_{E}\delta \right)+\sum_{k=2}^{\infty }\pi_{\left(k,1,0,1\right)}\left(1-p\right)\gamma_{d}\left(1-\delta \right)\\ \pi_{\left(1,0,0,0\right)}=\sum_{k=1}^{\infty }\pi_{\left(k,0,0,0\right)}p\gamma_{d}\left(1-\gamma_{E}\delta \right) \end{array}\right.$$

Each equation in (1) shows how the state vector on the left can be transferred to from the state vectors on the right under their corresponding conditions.

We denote $\Delta_{\delta }$ as the stationary AoI of the system when a probabilistic deletion/retaining scheme is applied. From the expectation formula, the average δ−secure AoI is calculated as

(2) $${\bar{\Delta }}_{\delta }=\sum_{n=1}^{\infty }n\cdot \mathrm{Pr}\left\{\Delta_{\delta }=n\right\}$$

where in general cases

(3) $$\mathrm{Pr}\left\{\Delta_{\delta }=n\right\}=\pi_{\left(n,0,0,0\right)}+\sum_{i=0}^{1}\sum_{m=1}^{n-1}\pi_{\left(n,m,0,i\right)}+\sum_{i=0}^{1}\sum_{l=1}^{n-2}\sum_{m=l+1}^{n-1}\pi_{\left(n,m,l,i\right)}$$

since the AoI is represented by the first component of the four-dimensional state vector.

Define the probability generation function $H_{\delta }\left(z\right)$ as

(4) $$\begin{array}{cc} H_{\delta }\left(z\right) & =\sum_{n=1}^{\infty }z^{n}\mathrm{Pr}\left\{\Delta_{\delta }=n\right\}\\ \; & =\sum_{n=1}^{\infty }z^{n}\pi_{\left(n,0,0,0\right)}+\sum_{i=0}^{1}\sum_{m=1}^{\infty }\sum_{n=m+1}^{\infty }z^{n}\pi_{\left(n,m,0,i\right)} \end{array}$$

(5) $$\begin{array}{cc} \; & \;+\sum_{i=0}^{1}\sum_{l=1}^{\infty }\sum_{m=l+1}^{\infty }\sum_{n=m+1}^{\infty }z^{n}\pi_{\left(n,m,l,i\right)} \end{array}$$

Equation (5) is obtained by substituting (3). It sees from (4) that

(6) $${\bar{\Delta }}_{\delta }={\left.\frac{dH_{\delta }\left(z\right)}{dz}\right|}_{z=1}$$

In the following paragraphs, we obtain the average δ−secure AoI by deriving $H_{\delta }\left(z\right)$ and calculating its derivative at z=1.

To obtain $H_{\delta }\left(z\right)$, we denote that

(7) $$\begin{array}{c} h_{1}\left(z\right)=\sum_{n=1}^{\infty }z^{n}\pi_{\left(n,0,0,0\right)} \end{array}$$

(8) $$\begin{array}{c} h_{2}\left(z\right)=\sum_{m=1}^{\infty }\sum_{n=m+1}^{\infty }z^{n}\pi_{\left(n,m,0,0\right)},\;h_{4}\left(z\right)=\sum_{l=1}^{\infty }\sum_{m=l+1}^{\infty }\sum_{n=m+1}^{\infty }z^{n}\pi_{\left(n,m,l,0\right)} \end{array}$$

(9) $$\begin{array}{c} h_{3}\left(z\right)=\sum_{m=1}^{\infty }\sum_{n=m+1}^{\infty }z^{n}\pi_{\left(n,m,0,1\right)},\;h_{5}\left(z\right)=\sum_{l=1}^{\infty }\sum_{m=l+1}^{\infty }\sum_{n=m+1}^{\infty }z^{n}\pi_{\left(n,m,l,1\right)} \end{array}$$

**Lemma 1.** 
*The functions defined in (7)–(9) satisfy the relationships that*

(10) $$\begin{array}{c} h_{3}\left(z\right)=\frac{p\left(1-\gamma_{d}\right)\gamma_{E}\left(1-\delta \right)z}{1-\left(1-p\right)\left(1-\gamma_{d}\right)\left(1-\delta \right)z}h_{1}\left(z\right)+\frac{\left(1-p\right)\left(1-\gamma_{d}\right)\gamma_{E}\left(1-\delta \right)z}{1-\left(1-p\right)\left(1-\gamma_{d}\right)\left(1-\delta \right)z}h_{2}\left(z\right) \end{array}$$

(11) $$\begin{array}{c} h_{4}\left(z\right)=\frac{p\left(1-\gamma_{d}\right)\left(1-\gamma_{E}\right)z}{1-\left(1-\gamma_{d}\right)\left(1-\gamma_{E}\right)z}h_{2}\left(z\right) \end{array}$$

(12) $$\begin{array}{c} h_{5}\left(z\right)=\frac{p\left(1-\gamma_{d}\right)\gamma_{E}\left(1-\delta \right)zh_{2}\left(z\right)}{\left[1-\left(1-\gamma_{d}\right)\left(1-\gamma_{E}\right)z\right]\left[1-\left(1-\gamma_{d}\right)\left(1-\delta \right)z\right]}+\frac{p\left(1-\gamma_{d}\right)\left(1-\delta \right)zh_{3}\left(z\right)}{1-\left(1-\gamma_{d}\right)\left(1-\delta \right)z} \end{array}$$

We prove Lemma 1 in Appendix A. Using Equations (10)–(12), it shows that

(13) $$\begin{array}{cc} H_{\delta }\left(z\right) & =h_{1}\left(z\right)+h_{2}\left(z\right)+h_{3}\left(z\right)+h_{4}\left(z\right)+h_{5}\left(z\right)=h_{1}\left(z\right)+h_{2}\left(z\right)+h_{3}\left(z\right)+\frac{p\left(1-\gamma_{d}\right)\left(1-\gamma_{E}\right)z}{1-\left(1-\gamma_{d}\right)\left(1-\gamma_{E}\right)z}h_{2}\left(z\right)\\ \; & \;+\frac{p\left(1-\gamma_{d}\right)\gamma_{E}\left(1-\delta \right)z}{\left[1-\left(1-\gamma_{d}\right)\left(1-\gamma_{E}\right)z\right]\left[1-\left(1-\gamma_{d}\right)\left(1-\delta \right)z\right]}h_{2}\left(z\right)+\frac{p\left(1-\gamma_{d}\right)\left(1-\delta \right)z}{1-\left(1-\gamma_{d}\right)\left(1-\delta \right)z}h_{3}\left(z\right)\\ \; & =h_{1}\left(z\right)+h_{2}\left(z\right)\left(\frac{1-\left(1-p\right)\left(1-\gamma_{d}\right)\left(1-\gamma_{E}\right)z}{1-\left(1-\gamma_{d}\right)\left(1-\gamma_{E}\right)z}+\frac{p\left(1-\gamma_{d}\right)\gamma_{E}\left(1-\delta \right)z}{\left[1-\left(1-\gamma_{d}\right)\left(1-\gamma_{E}\right)z\right]\left[1-\left(1-\gamma_{d}\right)\left(1-\delta \right)z\right]}\right)\\ \; & \;+\frac{1-\left(1-p\right)\left(1-\gamma_{d}\right)\left(1-\delta \right)z}{1-\left(1-\gamma_{d}\right)\left(1-\delta \right)z}\left(\frac{p\left(1-\gamma_{d}\right)\gamma_{E}\left(1-\delta \right)z}{1-\left(1-p\right)\left(1-\gamma_{d}\right)\left(1-\delta \right)z}h_{1}\left(z\right)+\frac{\left(1-p\right)\left(1-\gamma_{d}\right)\gamma_{E}\left(1-\delta \right)z}{1-\left(1-p\right)\left(1-\gamma_{d}\right)\left(1-\delta \right)z}h_{2}\left(z\right)\right)\\ \; & =\left(1+\frac{p\left(1-\gamma_{d}\right)\gamma_{E}\left(1-\delta \right)z}{1-\left(1-\gamma_{d}\right)\left(1-\delta \right)z}\right)h_{1}\left(z\right)+\frac{\left[1-\left(1-p\right)\left(1-\gamma_{d}\right)\left(1-\gamma_{E}\right)z\right]\left[1-\left(1-\gamma_{d}\right)\left(1-\gamma_{E}\right)\left(1-\delta \right)z\right]}{\left[1-\left(1-\gamma_{d}\right)\left(1-\gamma_{E}\right)z\right]\left[1-\left(1-\gamma_{d}\right)\left(1-\delta \right)z\right]}h_{2}\left(z\right) \end{array}$$

By calculating the derivative of (13) and letting z=1, the average δ−secure AoI is determined to be

(14) $$\begin{array}{cc} {\bar{\Delta }}_{\delta } & =\frac{p\left(1-\gamma_{d}\right)\gamma_{E}\left(1-\delta \right)}{{\left[1-\left(1-\gamma_{d}\right)\left(1-\delta \right)\right]}^{2}}M_{1}+\left(1+\frac{p\left(1-\gamma_{d}\right)\gamma_{E}\left(1-\delta \right)}{1-\left(1-\gamma_{d}\right)\left(1-\delta \right)}\right)\left[{\left.\frac{dh_{1}\left(z\right)}{dz}\right|}_{z=1}\right]\\ \; & \;+\left(\frac{p\left(1-\gamma_{d}\right)\left(1-\gamma_{E}\right)\left[1-\left(1-\gamma_{d}\right)\left(1-\gamma_{E}\right)\left(1-\delta \right)\right]}{{\left[1-\left(1-\gamma_{d}\right)\left(1-\gamma_{E}\right)\right]}^{2}\left[1-\left(1-\gamma_{d}\right)\left(1-\delta \right)\right]}+\frac{\left(1-\gamma_{d}\right)\gamma_{E}\left(1-\delta \right)\left[1-\left(1-p\right)\left(1-\gamma_{d}\right)\left(1-\gamma_{E}\right)\right]}{\left[1-\left(1-\gamma_{d}\right)\left(1-\gamma_{E}\right)\right]{\left[1-\left(1-\gamma_{d}\right)\left(1-\delta \right)\right]}^{2}}\right)M_{2}\\ \; & \;+\frac{\left[1-\left(1-p\right)\left(1-\gamma_{d}\right)\left(1-\gamma_{E}\right)\right]\left[1-\left(1-\gamma_{d}\right)\left(1-\gamma_{E}\right)\left(1-\delta \right)\right]}{\left[1-\left(1-\gamma_{d}\right)\left(1-\gamma_{E}\right)\right]\left[1-\left(1-\gamma_{d}\right)\left(1-\delta \right)\right]}\left[{\left.\frac{dh_{2}\left(z\right)}{dz}\right|}_{z=1}\right] \end{array}$$

in which we denote that $M_{1}=h_{1}\left(1\right)$, $M_{2}=h_{2}\left(1\right)$.

We determine $M_{1}$, $M_{2}$, $h_{1}^{\prime }\left(1\right)$, and $h_{2}^{\prime }\left(1\right)$ in the following Lemma 2.

**Lemma 2.** 
*The derivatives of $h_{1}\left(z\right)$ and $h_{2}\left(z\right)$ at z=1 are determined by equations*

(15) $$\left\{\begin{array}{c} \;a_{11}h_{1}^{\prime }\left(1\right)+a_{12}h_{2}^{\prime }\left(1\right)=c_{1}M_{1}+c_{2}M_{2}+c_{3}\left[{\left.\frac{dh_{2}^{\left(m\right)}\left(z\right)}{dz}\right|}_{z=1}\right]\\ a_{21}h_{1}^{\prime }\left(1\right)+a_{22}h_{2}^{\prime }\left(1\right)=d_{1}M_{1}+d_{2}M_{2}+d_{3}\left[{\left.\frac{dh_{2}^{\left(m\right)}\left(z\right)}{dz}\right|}_{z=1}\right] \end{array}\right.$$

*in which*

(16) $$\begin{array}{c} a_{11}=p\left(1-\frac{\gamma_{E}\delta }{1-\left(1-p\right)\left(1-\gamma_{d}\right)\left(1-\delta \right)}\right),\;a_{12}=-\frac{\left(1-p\right)\gamma_{E}\delta }{1-\left(1-p\right)\left(1-\gamma_{d}\right)\left(1-\delta \right)} \end{array}$$

(17) $$\begin{array}{c} c_{1}=\left(1-p\right)+p\gamma_{d}\left(1-\gamma_{E}\right)+\frac{p\gamma_{E}\left[1-\left(1-\gamma_{d}\right)\left(1-\delta \right)\right]}{{\left[1-\left(1-p\right)\left(1-\gamma_{d}\right)\left(1-\delta \right)\right]}^{2}} \end{array}$$

(18) $$\begin{array}{c} c_{2}=\left(1-p\right)\gamma_{d}\left(1-\gamma_{E}\right)+\frac{\left(1-p\right)\gamma_{E}\left[1-\left(1-\gamma_{d}\right)\left(1-\delta \right)\right]}{{\left[1-\left(1-p\right)\left(1-\gamma_{d}\right)\left(1-\delta \right)\right]}^{2}} \end{array}$$

(19) $$\begin{array}{c} c_{3}=\left(1-p\right)\gamma_{d}\left(1-\gamma_{E}\right)+\frac{\left(1-p\right)\gamma_{d}\gamma_{E}\left(1-\delta \right)}{1-\left(1-p\right)\left(1-\gamma_{d}\right)\left(1-\delta \right)} \end{array}$$

*and for the second equation,*

(20) $$a_{21}=-p\left(1-\gamma_{d}\right)\left(1-\gamma_{E}\right)+p\gamma_{E}\delta \left(\frac{1}{1-\left(1-p\right)\left(1-\gamma_{d}\right)\left(1-\delta \right)}-\frac{1}{1-\left(1-\gamma_{d}\right)\left(1-\delta \right)}\right)$$

(21) $$\begin{array}{cc} a_{22} & =1-\left(1-p\right)\left(1-\gamma_{d}\right)\left(1-\gamma_{E}\right)\\ \; & \;-\frac{p\gamma_{E}\delta }{1-\left(1-\gamma_{d}\right)\left(1-\delta \right)}\left(\frac{1}{1-\left(1-\gamma_{d}\right)\left(1-\gamma_{E}\right)}+\frac{\left(1-p\right)\left(1-\gamma_{d}\right)\left(1-\gamma_{E}\right)}{1-\left(1-p\right)\left(1-\gamma_{d}\right)\left(1-\gamma_{E}\right)}\right) \end{array}$$

(22) $$d_{1}=p\left(1-\gamma_{d}\right)\left(1-\gamma_{E}\right)+p\gamma_{E}\left(\frac{1}{1-\left(1-\gamma_{d}\right)\left(1-\delta \right)}-\frac{1-\left(1-\gamma_{d}\right)\left(1-\delta \right)}{{\left[1-\left(1-p\right)\left(1-\gamma_{d}\right)\left(1-\delta \right)\right]}^{2}}\right)$$

(23) $$\begin{array}{cc} d_{2} & =\left(1-p\right)\left(1-\gamma_{d}\right)\left(1-\gamma_{E}\right)+\frac{p[1-\left(1-p\right)\left(1-\gamma_{d}\right)\left(1-\delta \right)]}{\left[1-\left(1-\gamma_{d}\right)\left(1-\gamma_{E}\right)\right]\left[1-\left(1-\gamma_{d}\right)\left(1-\delta \right)\right]}\\ \; & \;+\frac{p\gamma_{E}\left(1-p\right)\left(1-\gamma_{d}\right)\left(1-\delta \right)}{1-\left(1-p\right)\left(1-\gamma_{d}\right)\left(1-\delta \right)}\left(\frac{1}{1-\left(1-\gamma_{d}\right)\left(1-\delta \right)}+\frac{1}{1-\left(1-p\right)\left(1-\gamma_{d}\right)\left(1-\delta \right)}\right) \end{array}$$

(24) $$d_{3}=\frac{p\gamma_{d}\left[1-\gamma_{E}\delta -\left(1-\gamma_{d}\right)\left(1-\gamma_{E}\right)\left(1-\delta \right)\right]}{\left[1-\left(1-\gamma_{d}\right)\left(1-\gamma_{E}\right)\right]\left[1-\left(1-\gamma_{d}\right)\left(1-\delta \right)\right]}+\frac{p\gamma_{d}\gamma_{E}\left(1-p\right)\left(1-\gamma_{d}\right){\left(1-\delta \right)}^{2}}{\left[1-\left(1-\gamma_{d}\right)\left(1-\delta \right)\right]\left[1-\left(1-p\right)\left(1-\gamma_{d}\right)\left(1-\delta \right)\right]}$$

*Define $h_{2}^{\left(m\right)}\left(z\right)=\sum_{m=1}^{\infty }\sum_{n=m+1}^{\infty }z^{m}\pi_{\left(n,m,0,0\right)}$, it proves that ${\left.dh_{2}^{\left(m\right)}\left(z\right)/dz\right|}_{z=1}$ is determined by*

(25) $$\begin{array}{cc} {\left.\frac{dh_{2}^{\left(m\right)}\left(z\right)}{dz}\right|}_{z=1} & =\frac{p\left(1-\gamma_{d}\right)\left(1-\gamma_{E}\right)+\frac{p^{2}\left(1-\gamma_{d}\right)\gamma_{E}\left(1-\delta \right)}{\left[1-\left(1-\gamma_{d}\right)\left(1-\delta \right)\right]\left[1-\left(1-p\right)\left(1-\gamma_{d}\right)\left(1-\delta \right)\right]}}{1-\left(1-p\right)\left(1-\gamma_{d}\right)\left(1-\gamma_{E}\right)}M_{1}\\ \; & +\frac{\left(1-p\right)\left(1-\gamma_{d}\right)\left(1-\gamma_{E}\right)+\frac{p[1-\left(1-\gamma_{d}\right)\left(1-\gamma_{E}\right)\left(1-\delta \right)]}{\left[1-\left(1-\gamma_{d}\right)\left(1-\gamma_{E}\right)\right]\left[1-\left(1-\gamma_{d}\right)\left(1-\delta \right)\right]}+\frac{p\left(1-p\right)\left(1-\gamma_{d}\right)\gamma_{E}\left(1-\delta \right)}{\left[1-\left(1-\gamma_{d}\right)\left(1-\delta \right)\right]\left[1-\left(1-p\right)\left(1-\gamma_{d}\right)\left(1-\delta \right)\right]}}{1-\left(1-p\right)\left(1-\gamma_{d}\right)\left(1-\gamma_{E}\right)}M_{2} \end{array}$$

*Denote*

(26) $$R_{1,2}=\frac{p\left(1-\gamma_{d}\right)\left(\left(1-\gamma_{E}\right)\left[1-\left(1-p\right)\left(1-\gamma_{d}\right)\left(1-\delta \right)\right]+p\gamma_{E}\left(1-\delta \right)\right)}{\left(1-p\right)\left(\left[1-\left(1-\gamma_{d}\right)\left(1-\gamma_{E}\right)\right]\left[1-\left(1-p\right)\left(1-\gamma_{d}\right)\left(1-\delta \right)\right]-p\left(1-\gamma_{d}\right)\gamma_{E}\left(1-\delta \right)\right)}$$

*then $M_{2}=R_{1,2}M_{1}$, and finally $M_{1}$ is given as*

(27) $$M_{1}={\left(1+\frac{p\left(1-\gamma_{d}\right)\gamma_{E}\left(1-\delta \right)}{1-\left(1-\gamma_{d}\right)\left(1-\delta \right)}+\frac{\left[1-\left(1-p\right)\left(1-\gamma_{d}\right)\left(1-\gamma_{E}\right)\right]\left[1-\left(1-\gamma_{d}\right)\left(1-\gamma_{E}\right)\left(1-\delta \right)\right]}{\left[1-\left(1-\gamma_{d}\right)\left(1-\gamma_{E}\right)\right]\left[1-\left(1-\gamma_{d}\right)\left(1-\delta \right)\right]}R_{1,2}\right)}^{-1}$$

Derivations of results (15)–(27) are given in Appendix B. Combining Lemma 2 and Equation (14), we derive average δ−secure AoI as follows.

**Theorem 1.** 
*Assuming that the packets transmitted through a Ber/Geo/1/2 queue modeled status updating system are eavesdropped with probability $\gamma_{E}$ in each time slot, when applying the probabilistic deletion/retaining transmission scheme, the average δ−secure AoI of the system is derived to be*

(28) $$\begin{array}{cc} {\bar{\Delta }}_{\delta } & =\frac{p\left(1-\gamma_{d}\right)\gamma_{E}\left(1-\delta \right)}{{\left[1-\left(1-\gamma_{d}\right)\left(1-\delta \right)\right]}^{2}}M_{1}+\frac{\left(e_{1}a_{22}-e_{2}a_{21}\right)c^{T}f-\left(e_{1}a_{12}-e_{2}a_{11}\right)d^{T}f}{a_{11}a_{22}-a_{12}a_{21}}\\ \; & \;+\left(\frac{p\left(1-\gamma_{d}\right)\left(1-\gamma_{E}\right)\left[1-\left(1-\gamma_{d}\right)\left(1-\gamma_{E}\right)\left(1-\delta \right)\right]}{{\left[1-\left(1-\gamma_{d}\right)\left(1-\gamma_{E}\right)\right]}^{2}\left[1-\left(1-\gamma_{d}\right)\left(1-\delta \right)\right]}+\frac{\left(1-\gamma_{d}\right)\gamma_{E}\left(1-\delta \right)\left[1-\left(1-p\right)\left(1-\gamma_{d}\right)\left(1-\gamma_{E}\right)\right]}{\left[1-\left(1-\gamma_{d}\right)\left(1-\gamma_{E}\right)\right]{\left[1-\left(1-\gamma_{d}\right)\left(1-\delta \right)\right]}^{2}}\right)M_{2} \end{array}$$

*where*

(29) $$c=\left(c_{1},c_{2},c_{3}\right),\;d=\left(d_{1},d_{2},d_{3}\right),\;f=\left(M_{1},M_{2},{\left.\frac{dh_{2}^{\left(m\right)}\left(z\right)}{dz}\right|}_{z=1}\right)$$

*the superscript T represents transposition. In Equation (28), we denote $e_{1}$ and $e_{2}$ to be*

(30) $$e_{1}=1+\frac{p\left(1-\gamma_{d}\right)\gamma_{E}\left(1-\delta \right)}{1-\left(1-\gamma_{d}\right)\left(1-\delta \right)},\;e_{2}=\frac{\left[1-\left(1-p\right)\left(1-\gamma_{d}\right)\left(1-\gamma_{E}\right)\right]\left[1-\left(1-\gamma_{d}\right)\left(1-\gamma_{E}\right)\left(1-\delta \right)\right]}{\left[1-\left(1-\gamma_{d}\right)\left(1-\gamma_{E}\right)\right]\left[1-\left(1-\gamma_{d}\right)\left(1-\delta \right)\right]}$$

**Proof.**  Denote that

$$c=\left(c_{1},c_{2},c_{3}\right),\;d=\left(d_{1},d_{2},d_{3}\right),\;f=\left(M_{1},M_{2},{\left.\frac{dh_{2}^{\left(m\right)}\left(z\right)}{dz}\right|}_{z=1}\right)$$

Equation (15) can be represented as

(31) $$\left[\begin{array}{cc} a_{11} & a_{12}\\ a_{21} & a_{22} \end{array}\right]\left[\begin{array}{c} h_{1}^{\prime }\left(1\right)\\ h_{2}^{\prime }\left(1\right) \end{array}\right]=\left[\begin{array}{c} c^{T}f\\ d^{T}f \end{array}\right]$$

from which we solve that

(32) $$\left[\begin{array}{c} h_{1}^{\prime }\left(1\right)\\ h_{2}^{\prime }\left(1\right) \end{array}\right]=\frac{1}{a_{11}a_{22}-a_{12}a_{21}}\left[\begin{array}{cc} a_{22} & -a_{12}\\ -a_{21} & a_{11} \end{array}\right]\left[\begin{array}{c} c^{T}f\\ d^{T}f \end{array}\right]$$

Define

$$e_{1}=1+\frac{p\left(1-\gamma_{d}\right)\gamma_{E}\left(1-\delta \right)}{1-\left(1-\gamma_{d}\right)\left(1-\delta \right)},\;e_{2}=\frac{\left[1-\left(1-p\right)\left(1-\gamma_{d}\right)\left(1-\gamma_{E}\right)\right]\left[1-\left(1-\gamma_{d}\right)\left(1-\gamma_{E}\right)\left(1-\delta \right)\right]}{\left[1-\left(1-\gamma_{d}\right)\left(1-\gamma_{E}\right)\right]\left[1-\left(1-\gamma_{d}\right)\left(1-\delta \right)\right]}$$

then,

(33) $$\begin{array}{cc} e_{1}h_{1}^{\prime }\left(1\right)+e_{2}h_{2}^{\prime }\left(1\right) & =\left[e_{1}\;\;e_{2}\right]\left[\begin{array}{c} h_{1}^{\prime }\left(1\right)\\ h_{2}^{\prime }\left(1\right) \end{array}\right]\\ \; & =\frac{1}{a_{11}a_{22}-a_{12}a_{21}}\left[e_{1}\;\;e_{2}\right]\left[\begin{array}{cc} a_{22} & -a_{12}\\ -a_{21} & a_{11} \end{array}\right]\left[\begin{array}{c} c^{T}f\\ d^{T}f \end{array}\right]\\ \; & =\frac{\left(e_{1}a_{22}-e_{2}a_{21}\right)c^{T}f-\left(e_{1}a_{12}-e_{2}a_{11}\right)d^{T}f}{a_{11}a_{22}-a_{12}a_{21}} \end{array}$$

Substituting (33) into (14) gives the average δ−secure AoI in Equation (28). This completes the proof of Theorem 1. □

The formula given in Theorem 1 is complex. With special case $\gamma_{E}=0$, we first verify that Equation (28) reduces to the average AoI of a basic Ber/Geo/1/2 queue modeled status updating system.

Letting $\gamma_{E}=0$ in Equations (26) and (27), it gives that

(34) $$\begin{array}{c} R_{1,2}=\frac{p(1-\gamma_{d})}{\left(1-p\right)\gamma_{d}} \end{array}$$

(35) $$\begin{array}{c} M_{1}=\frac{\left(1-p\right)\gamma_{d}^{2}}{{\left[1-\left(1-p\right)\left(1-\gamma_{d}\right)\right]}^{2}-p\gamma_{d}} \end{array}$$

(36) $$\begin{array}{c} M_{2}=\frac{p\gamma_{d}\left(1-\gamma_{d}\right)}{{\left[1-\left(1-p\right)\left(1-\gamma_{d}\right)\right]}^{2}-p\gamma_{d}} \end{array}$$

(37) $$\begin{array}{c} M_{3}=0 \end{array}$$

in which (37) is obtained by referring to Equation (A27).

Substituting $\gamma_{E}=0$ and $M_{3}=0$ into (A23), we have

(38) $$h_{2}^{\left(m\right)}\left(z\right)\left[1-\left(1-p\right)\left(1-\gamma_{d}\right)z\right]=p\left(1-\gamma_{d}\right)M_{1}z+\frac{p\gamma_{d}M_{2}z}{1-(1-\gamma_{d})z}$$

Since $M_{2}=R_{1,2}M_{1}$, that is $p\left(1-\gamma_{d}\right)M_{1}=\left(1-p\right)\gamma_{d}M_{2}$, from (38), we obtain that

(39) $$h_{2}^{\left(m\right)}\left(z\right)=\frac{\gamma_{d}M_{2}z}{1-(1-\gamma_{d})z}$$

In the case of $\gamma_{E}=0$, Equations (A15) and (A16) are simplified to

(40) $$\begin{array}{c} h_{1}\left(z\right)=\frac{p\gamma_{d}M_{1}z}{1-(1-p)z}+\frac{\left(1-p\right)\gamma_{d}z}{1-(1-p)z}h_{2}^{\left(m\right)}\left(z\right) \end{array}$$

(41) $$\begin{array}{c} h_{2}\left(z\right)=\frac{p(1-\gamma_{d})z}{1-\left(1-p\right)\left(1-\gamma_{d}\right)z}h_{1}\left(z\right)+\frac{p\gamma_{d}z}{\left[1-\left(1-\gamma_{d}\right)z\right]\left[1-\left(1-p\right)\left(1-\gamma_{d}\right)z\right]}h_{2}^{\left(m\right)}\left(z\right) \end{array}$$

Combining (13), (40), (41), and (38), one can calculate that

(42) $$\begin{array}{cc} H_{\delta }\left(z\right) & =h_{1}\left(z\right)+\frac{1-\left(1-p\right)\left(1-\gamma_{d}\right)z}{1-(1-\gamma_{d})z}h_{2}\left(z\right)\\ \; & =\frac{p\gamma_{d}M_{1}z\left[1-\left(1-p\right)\left(1-\gamma_{d}\right)z\right]}{\left[1-\left(1-p\right)z\right]{\left[1-\left(1-\gamma_{d}\right)z\right]}^{2}}+\frac{p\gamma_{d}^{2}M_{2}z^{2}}{{\left[1-\left(1-\gamma_{d}\right)z\right]}^{3}} \end{array}$$

Finally, through direct calculation,

(43) $$\begin{array}{cc} {\left.\frac{dH_{\delta }\left(z\right)}{dz}\right|}_{z=1} & =\frac{{\left[1-\left(1-p\right)\left(1-\gamma_{d}\right)\right]}^{2}}{p\gamma_{d}^{2}}M_{1}+\frac{\left[1-\left(1-p\right)\left(1-\gamma_{d}\right)\right]+2p\left(1-\gamma_{d}\right)}{\gamma_{d}^{2}}M_{2} \end{array}$$

(44) $$\begin{array}{cc} \; & =\frac{1}{\gamma_{d}}\left(\left(1-\gamma_{d}\right)+\frac{1}{\rho_{d}}+\frac{2\rho_{d}^{2}{\left(1-\gamma_{d}\right)}^{2}}{1+\rho_{d}\left(1-2\gamma_{d}\right)+\rho_{d}^{2}{\left(1-\gamma_{d}\right)}^{2}}\right) \end{array}$$

(45) $$\begin{array}{cc} \; & ={\bar{\Delta }}_{Ber/Geo/1/2} \end{array}$$

where in (44), we denote $\rho_{d}=p/\gamma_{d}$ as the discrete traffic intensity. Equation (44) is exactly the average AoI of the status updating system that uses the Ber/Geo/1/2 queue, which was determined in our previous work [29].

Notice that when $\gamma_{E}=0$, all of the packets that are sent out from transmitter will never be obtained by the eavesdropper. As a result, the probabilistic deletion/retaining is never triggered, and all the packets are transmitted to the receiver securely. In this case, it is proved that average δ−secure AoI degenerates to ${\bar{\Delta }}_{Ber/Geo/1/2}$. Since the probabilistic deletion/retaining scheme is never performed, it is observed that deletion probability δ disappears in Equations (34)–(41), and finally is not contained in the average AoI formula.

We use average AoI to characterize packet freshness, while the transmission security of the system is measured by the proportion of insecure packets that the receiver obtains. In following theorem, we determine this insecure packet proportion under a probabilistic deletion/retaining transmission scheme.

**Theorem 2.** 
*Let the deletion probability be δ. When applying the probabilistic deletion/retaining scheme to transmit packets over the Ber/Geo/1/2 queue modeled status updating system, the probability that an insecure packet arrives at the receiver, as well as the proportion of insecure packets that the receiver obtains over a long period of time, which is denoted as η(δ), is determined to be*

(46) $$\eta \left(\delta \right)=\frac{\gamma_{d}\gamma_{E}\left(1-\delta \right)}{\left[1-\left(1-\gamma_{d}\right)\left(1-\gamma_{E}\right)\right]\left[1-\left(1-\gamma_{d}\right)\left(1-\delta \right)\right]}$$

**Proof.**  Define three events as

$$\begin{array}{cc} \; & E:\mathrm{an}\;\mathrm{insecure}\;\mathrm{packet}\;\mathrm{arrives}\;\mathrm{at}\;\mathrm{the}\;\mathrm{receiver}\\ \; & E_{1}:a\;\mathrm{transmitted}\;\mathrm{packet}\;\mathrm{becomes}\;\mathrm{insecure}\\ \; & E_{2}:\mathrm{an}\;\mathrm{insecure}\;\mathrm{packet}\;\mathrm{arrives}\;\mathrm{at}\;d\;\mathrm{before}\;\mathrm{being}\;\mathrm{deleted} \end{array}$$

Then,

(47) $$\mathrm{Pr}\left(E\right)=\eta \left(\delta \right)=\mathrm{Pr}\left(E_{1}\right)\cdot \mathrm{Pr}\left(E_{2}\right)$$

Let $T_{d}$, $T_{E}$, and $T_{\delta }$ be the time that a packet is transmitted to *d*, *E*, and that of being deleted under the probabilistic deletion/retaining scheme. Event $E_{1}$ requires that $T_{d}\geq T_{E}$, while event $E_{2}$ needs $T_{\delta }>T_{d}$. Due to the memoryless property of geometric distributions, these two events are independent. Therefore,

(48) $$\begin{array}{cc} \eta (\delta )=\mathrm{Pr}(E) & =\mathrm{Pr}\left\{T_{d}\geq T_{E}\right\}\cdot \mathrm{Pr}\left\{T_{\delta }>T_{d}\right\}\\ \; & =\left(\sum_{j=1}^{\infty }\mathrm{Pr}\left\{T_{E}=j\right\}\mathrm{Pr}\left\{T_{d}\geq j\right\}\right)\left(\sum_{l=1}^{\infty }\mathrm{Pr}\left\{T_{d}=l\right\}\mathrm{Pr}\left\{T_{\delta }>l\right\}\right)\\ \; & =\left(\sum_{j=1}^{\infty }{\left(1-\gamma_{E}\right)}^{j-1}\gamma_{E}\sum_{k=j}^{\infty }{\left(1-\gamma_{d}\right)}^{k-1}\gamma_{d}\right)\left(\sum_{l=1}^{\infty }{\left(1-\gamma_{d}\right)}^{l-1}\gamma_{d}\sum_{k=l+1}^{\infty }{\left(1-\delta \right)}^{k-1}\delta \right)\\ \; & =\frac{\gamma_{d}\gamma_{E}\left(1-\delta \right)}{\left[1-\left(1-\gamma_{d}\right)\left(1-\gamma_{E}\right)\right]\left[1-\left(1-\gamma_{d}\right)\left(1-\delta \right)\right]} \end{array}$$

This derives the results in Theorem 2. □

For the given deletion probability δ, ${\bar{\Delta }}_{\delta }$ characterizes the freshness of the obtained packets, while η(δ), i.e., the proportion of insecure packets that are transmitted to the receiver, can be viewed as the security measurement of the system. Combining Theorem 1 and Theorem 2, and by introducing deletion probability δ, we actually establish the relationships between the timeliness performance and security performance of the considered status updating system, which enables us to investigate the tradeoffs between these two performance types.

## 4. Packet with Random Service Time Deadline and Average Age of Secure Information

Before investigating the relationships between ${\bar{\Delta }}_{\delta }$, η(δ) and system parameters *p*, $\gamma_{d}$, and $\gamma_{E}$, in this section we further consider two special cases of Equation (28) by setting $\gamma_{E}=1$ and δ=1. We explain that $\gamma_{E}=1$ corresponds to the situation with the packet with geometric deadline in service process. When δ=1, the average δ−secure AoI is equal to the average age of secure information (AoSI) that was studied in works [39,40] for bufferless systems.

### 4.1. Packet with Random Geometric Deadline in Service Process: $\gamma_{E}=1$

Assuming that $\gamma_{E}=1$, then in every time slot, each packet that enters the server will be obtained by an eavesdropper. This is equivalent to saying that each transmitted packet is insecure and a probabilistic deletion/retaining scheme will be performed in every time slot as long as there is a packet in service. With probability δ, the packet is deleted, and with probability 1−δ, this packet is retained. Observing that this transmission situation is exactly the scenario of transmitting packets under the restriction that the packets are controlled by the geometric deadline in service process, in this subsection, we derive the explicit formula of average AoI.

Letting $\gamma_{E}=1$, the main results required to derive ${\bar{\Delta }}_{\delta }$ are simplified to be

(49) $$\begin{array}{c} R_{1,2}=\frac{p^{2}\left(1-\gamma_{d}\right)\left(1-\delta \right)}{\left(1-p\right)[1-\left(1-\gamma_{d}\right)\left(1-\delta \right)]} \end{array}$$

(50) $$\begin{array}{c} M_{1}=\frac{\left(1-p\right){\left[1-\left(1-\gamma_{d}\right)\left(1-\delta \right)\right]}^{2}}{\left(1-p\right)\left[1-\left(1-\gamma_{d}\right)\left(1-\delta \right)\right]\left[1-\left(1-p\right)\left(1-\gamma_{d}\right)\left(1-\delta \right)\right]+p^{2}\left(1-\gamma_{d}\right)\left(1-\delta \right)} \end{array}$$

(51) $$\begin{array}{c} M_{3}=\frac{p\left(1-\gamma_{d}\right)\left(1-\delta \right)}{1-\left(1-\gamma_{d}\right)\left(1-\delta \right)}M_{1}=\frac{1-p}{p}M_{2} \end{array}$$

(52) $$\begin{array}{c} h_{2}^{\left(m\right)}\left(z\right)=\frac{\left[1-\left(1-\gamma_{d}\right)\left(1-\delta \right)\right]M_{2}z}{1-\left(1-\gamma_{d}\right)\left(1-\delta \right)z} \end{array}$$

(53) $$\begin{array}{c} H_{\delta }\left(z\right)=\left(1+\frac{p\left(1-\gamma_{d}\right)\left(1-\delta \right)z}{1-\left(1-\gamma_{d}\right)\left(1-\delta \right)z}\right)h_{1}\left(z\right)+\frac{h_{2}\left(z\right)}{1-\left(1-\gamma_{d}\right)\left(1-\delta \right)z} \end{array}$$

and

(54) $$\begin{array}{cc} \; & \left(\left[1-\left(1-p\right)z\right]\left[1-\left(1-p\right)\left(1-\gamma_{d}\right)\left(1-\delta \right)z\right]-p\delta z\right)h_{1}\left(z\right)\\ \; & \;=\left(1-p\right)\delta zh_{2}\left(z\right)+p\gamma_{d}\left(1-\delta \right)M_{1}z+\left(1-p\right)\gamma_{d}\left(1-\delta \right)zh_{2}^{\left(m\right)}\left(z\right)\\ \; & \left(\left[1-\left(1-\gamma_{d}\right)\left(1-\delta \right)z\right]\left[1-\left(1-p\right)\left(1-\gamma_{d}\right)\left(1-\delta \right)z\right]-p\delta z\right)h_{2}\left(z\right) \end{array}$$

(55) $$\begin{array}{cc} \; & \;=p^{2}\delta \left(1-\gamma_{d}\right)\left(1-\delta \right)z^{2}h_{1}\left(z\right)+p^{2}\gamma_{d}\left(1-\gamma_{d}\right){\left(1-\delta \right)}^{2}M_{1}z^{2}+p\gamma_{d}\left(1-\delta \right)zh_{2}^{\left(m\right)}\left(z\right) \end{array}$$

From Equations (49)–(55), we have the following theorem.

**Theorem 3.** 
*For the case where packets are restricted by a random geometric deadline during service process, the average AoI of Ber/Geo/1/2 queue modeled status updating system is determined as*

(56) $${\bar{\Delta }}_{\delta }=\frac{\left[1-\left(1-p\right)\left(1-\gamma_{d}\right)\left(1-\delta \right)\right]h_{1}^{\prime }\left(1\right)+h_{2}^{\prime }\left(1\right)}{1-\left(1-\gamma_{d}\right)\left(1-\delta \right)}+\frac{\left(1-\gamma_{d}\right)\left(1-\delta \right)\left(pM_{1}+M_{2}\right)}{{\left[1-\left(1-\gamma_{d}\right)\left(1-\delta \right)\right]}^{2}}$$

*in which δ is the expiration probability in each time slot. Two numbers $M_{1}$ and $M_{2}$ are given in (49) and (50) by noting that $M_{2}=R_{1,2}M_{1}$, and $h_{1}^{\prime }\left(1\right)$, $h_{2}^{\prime }\left(1\right)$ are obtained by*

(57) $$\begin{array}{cc} h_{1}^{\prime }\left(1\right) & =(\frac{p^{2}\delta^{2}\left(1-\gamma_{d}\right)\left(1-\delta \right)\left[1-\left(1-p\right)\left(1-\gamma_{d}\right)\right]N}{\left[1-\left(1-p\right)\left(1-\gamma_{d}\right)\left(1-\delta \right)\right]\left(\left[1-\left(1-p\right)\left(1-\gamma_{d}\right)\right]\left[1-\left(1-\gamma_{d}\right)\left(1-\delta \right)\right]-p\delta \right)}\\ \; & \;+\frac{\left[1-\left(1-\gamma_{d}\right)\left(1-\delta \right)\right]\left[1-{\left(1-p\right)}^{2}\left(1-\gamma_{d}\right)\left(1-\delta \right)\right]}{p^{2}\left(1-\gamma_{d}\right)\left(1-\delta \right)}+\frac{\gamma_{d}\left(1-\delta \right)}{1-\left(1-\gamma_{d}\right)\left(1-\delta \right)})\frac{\left(1-p\right)M_{2}}{p\left(1-\delta \right)[1-\left(1-p\right)\left(1-\gamma_{d}\right)]} \end{array}$$

*and*

(58) $$h_{2}^{\prime }\left(1\right)=\frac{p^{2}\delta \left(1-\gamma_{d}\right)\left(1-\delta \right)\left[1-\left(1-p\right)\left(1-\gamma_{d}\right)\right]N}{\left[1-\left(1-p\right)\left(1-\gamma_{d}\right)\left(1-\delta \right)\right]\left(\left[1-\left(1-p\right)\left(1-\gamma_{d}\right)\right]\left[1-\left(1-\gamma_{d}\right)\left(1-\delta \right)\right]-p\delta \right)}M_{2}$$

*in which N represents that*

(59) $$\begin{array}{cc} N & =\frac{\frac{\left(1-p\right)\left[1-\left(1-\gamma_{d}\right)\left(1-\delta \right)\right]\left[1-{\left(1-p\right)}^{2}\left(1-\gamma_{d}\right)\left(1-\delta \right)\right]}{p^{2}\left(1-\gamma_{d}\right)\left(1-\delta \right)}+\frac{\left(1-p\right)\gamma_{d}\left(1-\delta \right)}{1-\left(1-\gamma_{d}\right)\left(1-\delta \right)}}{p\left(1-\delta \right)[1-\left(1-p\right)\left(1-\gamma_{d}\right)]}\\ \; & \;+\frac{1-\left(1-p\right){\left(1-\gamma_{d}\right)}^{2}{\left(1-\delta \right)}^{2}-\left(1-p\right){\left[1-\left(1-\gamma_{d}\right)\left(1-\delta \right)\right]}^{2}+\frac{p\gamma_{d}\left(1-\delta \right)}{1-\left(1-\gamma_{d}\right)\left(1-\delta \right)}}{p^{2}\delta \left(1-\gamma_{d}\right)\left(1-\delta \right)} \end{array}$$

**Proof.**  Calculating the derivative of (53) and letting z=1, we have

(60) $$\begin{array}{cc} {\bar{\Delta }}_{\delta } & ={\left.\frac{dH_{\delta }\left(z\right)}{dz}\right|}_{z=1}\\ \; & =\frac{p\left(1-\gamma_{d}\right)\left(1-\delta \right)}{{\left[1-\left(1-\gamma_{d}\right)\left(1-\delta \right)\right]}^{2}}M_{1}+\frac{1-\left(1-p\right)\left(1-\gamma_{d}\right)\left(1-\delta \right)}{1-\left(1-\gamma_{d}\right)\left(1-\delta \right)}h_{1}^{\prime }\left(1\right)\\ \; & \;+\frac{\left(1-\gamma_{d}\right)\left(1-\delta \right)}{{\left[1-\left(1-\gamma_{d}\right)\left(1-\delta \right)\right]}^{2}}M_{2}+\frac{h_{2}^{\prime }\left(1\right)}{1-\left(1-\gamma_{d}\right)\left(1-\delta \right)}\\ \; & =\frac{\left[1-\left(1-p\right)\left(1-\gamma_{d}\right)\left(1-\delta \right)\right]h_{1}^{\prime }\left(1\right)+h_{2}^{\prime }\left(1\right)}{1-\left(1-\gamma_{d}\right)\left(1-\delta \right)}+\frac{\left(1-\gamma_{d}\right)\left(1-\delta \right)\left(pM_{1}+M_{2}\right)}{{\left[1-\left(1-\gamma_{d}\right)\left(1-\delta \right)\right]}^{2}} \end{array}$$

This derives the average AoI in (56). Since $M_{1}$ and $M_{2}$ were obtained in Equations (49) and (50), the remaining work to determine ${\bar{\Delta }}_{\delta }$ is obtaining $h_{1}^{\prime }\left(1\right)$ and $h_{2}^{\prime }\left(1\right)$.

Taking the derivative of both sides of (54) and (55), it proves that

(61) $$\begin{array}{cc} \; & p\left(1-\delta \right)\left[1-\left(1-p\right)\left(1-\gamma_{d}\right)\right]h_{1}^{\prime }\left(1\right)-\left(1-p\right)\delta h_{2}^{\prime }\left(1\right)\\ \; & \;=\left(\frac{\left(1-p\right)\left[1-\left(1-\gamma_{d}\right)\left(1-\delta \right)\right]\left[1-{\left(1-p\right)}^{2}\left(1-\gamma_{d}\right)\left(1-\delta \right)\right]}{p^{2}\left(1-\gamma_{d}\right)\left(1-\delta \right)}+\frac{\left(1-p\right)\gamma_{d}\left(1-\delta \right)}{1-\left(1-\gamma_{d}\right)\left(1-\delta \right)}\right)M_{2} \end{array}$$

and

$$\begin{array}{cc} \; & -p^{2}\delta \left(1-\gamma_{d}\right)\left(1-\delta \right)h_{1}^{\prime }\left(1\right)+\left(\left[1-\left(1-\gamma_{d}\right)\left(1-\delta \right)\right]\left[1-\left(1-p\right)\left(1-\gamma_{d}\right)\left(1-\delta \right)\right]-p\delta \right)h_{2}^{\prime }\left(1\right) \end{array}$$

(62) $$\begin{array}{c} \;=\left(1-\left(1-p\right){\left(1-\gamma_{d}\right)}^{2}{\left(1-\delta \right)}^{2}-\left(1-p\right){\left[1-\left(1-\gamma_{d}\right)\left(1-\delta \right)\right]}^{2}+\frac{p\gamma_{d}\left(1-\delta \right)}{1-\left(1-\gamma_{d}\right)\left(1-\delta \right)}\right)M_{2} \end{array}$$

Notice that during the calculations, we substitute

(63) $${\left.\frac{dh_{2}^{\left(m\right)}\left(z\right)}{dz}\right|}_{z=1}=\frac{M_{2}}{1-\left(1-\gamma_{d}\right)\left(1-\delta \right)}$$

which is obtained directly from Equation (52).

Writing (61) as

(64) $$\begin{array}{cc} \; & h_{1}^{\prime }\left(1\right)-\frac{(1-p)\delta }{p\left(1-\delta \right)[1-\left(1-p\right)\left(1-\gamma_{d}\right)]}h_{2}^{\prime }\left(1\right)=\frac{\frac{\left(1-p\right)\left[1-\left(1-\gamma_{d}\right)\left(1-\delta \right)\right]\left[1-{\left(1-p\right)}^{2}\left(1-\gamma_{d}\right)\left(1-\delta \right)\right]}{p^{2}\left(1-\gamma_{d}\right)\left(1-\delta \right)}+\frac{\left(1-p\right)\gamma_{d}\left(1-\delta \right)}{1-\left(1-\gamma_{d}\right)\left(1-\delta \right)}}{p\left(1-\delta \right)[1-\left(1-p\right)\left(1-\gamma_{d}\right)]}M_{2} \end{array}$$

and Equation (62) is equivalent to

$$\begin{array}{cc} \; & \frac{\left[1-\left(1-\gamma_{d}\right)\left(1-\delta \right)\right]\left[1-\left(1-p\right)\left(1-\gamma_{d}\right)\left(1-\delta \right)\right]-p\delta }{p^{2}\delta \left(1-\gamma_{d}\right)\left(1-\delta \right)}h_{2}^{\prime }\left(1\right)-h_{1}^{\prime }\left(1\right) \end{array}$$

(65) $$\begin{array}{c} \;=\frac{1-\left(1-p\right){\left(1-\gamma_{d}\right)}^{2}{\left(1-\delta \right)}^{2}-\left(1-p\right){\left[1-\left(1-\gamma_{d}\right)\left(1-\delta \right)\right]}^{2}+\frac{p\gamma_{d}\left(1-\delta \right)}{1-\left(1-\gamma_{d}\right)\left(1-\delta \right)}}{p^{2}\delta \left(1-\gamma_{d}\right)\left(1-\delta \right)}M_{2} \end{array}$$

From (64) and (65), we have

$$\begin{array}{cc} \; & h_{2}^{\prime }\left(1\right)\left(\frac{\left[1-\left(1-\gamma_{d}\right)\left(1-\delta \right)\right]\left[1-\left(1-p\right)\left(1-\gamma_{d}\right)\left(1-\delta \right)\right]-p\delta }{p^{2}\delta \left(1-\gamma_{d}\right)\left(1-\delta \right)}-\frac{(1-p)\delta }{p\left(1-\delta \right)[1-\left(1-p\right)\left(1-\gamma_{d}\right)]}\right) \end{array}$$

(66) $$\begin{array}{cc} = & h_{2}^{\prime }\left(1\right)\frac{\left[1-\left(1-p\right)\left(1-\gamma_{d}\right)\left(1-\delta \right)\right]\left(\left[1-\left(1-p\right)\left(1-\gamma_{d}\right)\right]\left[1-\left(1-\gamma_{d}\right)\left(1-\delta \right)\right]-p\delta \right)}{p^{2}\delta \left(1-\gamma_{d}\right)\left(1-\delta \right)\left[1-\left(1-p\right)\left(1-\gamma_{d}\right)\right]}\\ = & (\frac{\frac{\left(1-p\right)\left[1-\left(1-\gamma_{d}\right)\left(1-\delta \right)\right]\left[1-{\left(1-p\right)}^{2}\left(1-\gamma_{d}\right)\left(1-\delta \right)\right]}{p^{2}\left(1-\gamma_{d}\right)\left(1-\delta \right)}+\frac{\left(1-p\right)\gamma_{d}\left(1-\delta \right)}{1-\left(1-\gamma_{d}\right)\left(1-\delta \right)}}{p\left(1-\delta \right)[1-\left(1-p\right)\left(1-\gamma_{d}\right)]} \end{array}$$

(67) $$\begin{array}{cc} & \;+\frac{1-\left(1-p\right){\left(1-\gamma_{d}\right)}^{2}{\left(1-\delta \right)}^{2}-\left(1-p\right){\left[1-\left(1-\gamma_{d}\right)\left(1-\delta \right)\right]}^{2}+\frac{p\gamma_{d}\left(1-\delta \right)}{1-\left(1-\gamma_{d}\right)\left(1-\delta \right)}}{p^{2}\delta \left(1-\gamma_{d}\right)\left(1-\delta \right)})M_{2} \end{array}$$

Denote

(68) $$\begin{array}{cc} N & =\frac{\frac{\left(1-p\right)\left[1-\left(1-\gamma_{d}\right)\left(1-\delta \right)\right]\left[1-{\left(1-p\right)}^{2}\left(1-\gamma_{d}\right)\left(1-\delta \right)\right]}{p^{2}\left(1-\gamma_{d}\right)\left(1-\delta \right)}+\frac{\left(1-p\right)\gamma_{d}\left(1-\delta \right)}{1-\left(1-\gamma_{d}\right)\left(1-\delta \right)}}{p\left(1-\delta \right)[1-\left(1-p\right)\left(1-\gamma_{d}\right)]}\\ \; & \;+\frac{1-\left(1-p\right){\left(1-\gamma_{d}\right)}^{2}{\left(1-\delta \right)}^{2}-\left(1-p\right){\left[1-\left(1-\gamma_{d}\right)\left(1-\delta \right)\right]}^{2}+\frac{p\gamma_{d}\left(1-\delta \right)}{1-\left(1-\gamma_{d}\right)\left(1-\delta \right)}}{p^{2}\delta \left(1-\gamma_{d}\right)\left(1-\delta \right)} \end{array}$$

then Equation (67) shows that

(69) $$h_{2}^{\prime }\left(1\right)=\frac{p^{2}\delta \left(1-\gamma_{d}\right)\left(1-\delta \right)\left[1-\left(1-p\right)\left(1-\gamma_{d}\right)\right]N}{\left[1-\left(1-p\right)\left(1-\gamma_{d}\right)\left(1-\delta \right)\right]\left(\left[1-\left(1-p\right)\left(1-\gamma_{d}\right)\right]\left[1-\left(1-\gamma_{d}\right)\left(1-\delta \right)\right]-p\delta \right)}M_{2}$$

which is then combined with (64) to determine that

(70) $$\begin{array}{cc} h_{1}^{\prime }\left(1\right) & =(\frac{p^{2}\delta^{2}\left(1-\gamma_{d}\right)\left(1-\delta \right)\left[1-\left(1-p\right)\left(1-\gamma_{d}\right)\right]N}{\left[1-\left(1-p\right)\left(1-\gamma_{d}\right)\left(1-\delta \right)\right]\left(\left[1-\left(1-p\right)\left(1-\gamma_{d}\right)\right]\left[1-\left(1-\gamma_{d}\right)\left(1-\delta \right)\right]-p\delta \right)}\\ \; & +\frac{\left[1-\left(1-\gamma_{d}\right)\left(1-\delta \right)\right]\left[1-{\left(1-p\right)}^{2}\left(1-\gamma_{d}\right)\left(1-\delta \right)\right]}{p^{2}\left(1-\gamma_{d}\right)\left(1-\delta \right)}+\frac{\gamma_{d}\left(1-\delta \right)}{1-\left(1-\gamma_{d}\right)\left(1-\delta \right)})\frac{\left(1-p\right)M_{2}}{p\left(1-\delta \right)[1-\left(1-p\right)\left(1-\gamma_{d}\right)]} \end{array}$$

So far, both $h_{1}^{\prime }\left(1\right)$ and $h_{2}^{\prime }\left(1\right)$ are obtained. Thus, the proof is completed. □

### 4.2. Average Age of Secure Information: δ=1

Different from $\gamma_{E}=1$ in Section 4.1, in the second case we let δ=1. This means the transmitter will delete one packet immediately upon it becoming insecure. It is interesting to compare the differences in the impact on packets caused by controlling strategies in these two cases. Notice that if $\gamma_{E}=1$, then packet deletion is performed with probability δ in each time slot. However, in the case of δ=1, the packet is deleted with probability 1 but is not performed in every time slot, i.e., only in time slots when the served packet is insecure. The average AoI derived in the δ=1 case is exactly the average age of secure information (AoSI) of the Ber/Geo/1/2 queue modeled status updating system.

Like in Section 4.1, we start the analysis from a series of simplified results. By setting δ=1, we show that

(71) $$\begin{array}{c} M_{1}=\frac{\left(1-p\right){\left[1-\left(1-\gamma_{d}\right)\left(1-\gamma_{E}\right)\right]}^{2}}{\left(1-p\right){\left[1-\left(1-\gamma_{d}\right)\left(1-\gamma_{E}\right)\right]}^{2}+p\left(1-\gamma_{d}\right)\left(1-\gamma_{E}\right)\left[1-\left(1-p\right)\left(1-\gamma_{d}\right)\left(1-\gamma_{E}\right)\right]} \end{array}$$

(72) $$\begin{array}{c} M_{2}=\frac{p\left(1-\gamma_{d}\right)\left(1-\gamma_{E}\right)\left[1-\left(1-\gamma_{d}\right)\left(1-\gamma_{E}\right)\right]}{\left(1-p\right){\left[1-\left(1-\gamma_{d}\right)\left(1-\gamma_{E}\right)\right]}^{2}+p\left(1-\gamma_{d}\right)\left(1-\gamma_{E}\right)\left[1-\left(1-p\right)\left(1-\gamma_{d}\right)\left(1-\gamma_{E}\right)\right]} \end{array}$$

(73) $$\begin{array}{c} M_{3}=0 \end{array}$$

(74) $$\begin{array}{c} h_{2}^{\left(m\right)}\left(z\right)=\frac{\left[1-\left(1-\gamma_{d}\right)\left(1-\gamma_{E}\right)\right]M_{2}z}{1-\left(1-\gamma_{d}\right)\left(1-\gamma_{E}\right)z} \end{array}$$

(75) $$\begin{array}{c} H_{S}\left(z\right)=h_{1}\left(z\right)+\left(1+\frac{p\left(1-\gamma_{d}\right)\left(1-\gamma_{E}\right)z}{1-\left(1-\gamma_{d}\right)\left(1-\gamma_{E}\right)z}\right)h_{2}\left(z\right) \end{array}$$

(76) $$\begin{array}{c} \left(1-\left(1-p\right)z-p\gamma_{E}z\right)h_{1}\left(z\right)=\left(1-p\right)\gamma_{E}zh_{2}\left(z\right)+p\gamma_{d}\left(1-\gamma_{E}\right)M_{1}z+\left(1-p\right)\gamma_{d}\left(1-\gamma_{E}\right)zh_{2}^{\left(m\right)}\left(z\right) \end{array}$$

and

(77) $$\begin{array}{cc} \; & h_{2}\left(z\right)\left(1-\left(1-p\right)\left(1-\gamma_{d}\right)\left(1-\gamma_{E}\right)z-\frac{p\gamma_{E}z}{1-\left(1-\gamma_{d}\right)\left(1-\gamma_{E}\right)z}\right)\\ \; & \;\;=p\left(1-\gamma_{d}\right)\left(1-\gamma_{E}\right)zh_{1}\left(z\right)+\frac{p\gamma_{d}\left(1-\gamma_{E}\right)z}{1-\left(1-\gamma_{d}\right)\left(1-\gamma_{E}\right)z}h_{2}^{\left(m\right)}\left(z\right) \end{array}$$

**Theorem 4.** 
*For the Ber/Geo/1/2 queue modeled status updating system, assuming that with probability $\gamma_{E}$, a transmitted packet is eavesdropped in each time slot. Then, the average age of secure information, ${\bar{\Delta }}_{S}$, is obtained to be*

(78) $${\bar{\Delta }}_{S}=\frac{p\left(1-\gamma_{d}\right)\left(1-\gamma_{E}\right)}{{\left[1-\left(1-\gamma_{d}\right)\left(1-\gamma_{E}\right)\right]}^{2}}M_{2}+h_{1}^{\prime }\left(1\right)+\left(1+\frac{p\left(1-\gamma_{d}\right)\left(1-\gamma_{E}\right)}{1-\left(1-\gamma_{d}\right)\left(1-\gamma_{E}\right)}\right)h_{2}^{\prime }\left(1\right)$$

*in which*

(79) $$\begin{array}{cc} h_{1}^{\prime }\left(1\right) & =\frac{\left(1-\left(1-p\right)\left(1-\gamma_{d}\right)\left(1-\gamma_{E}\right)-\frac{p\gamma_{E}}{1-\left(1-\gamma_{d}\right)\left(1-\gamma_{E}\right)}\right)\left(1+\frac{p\gamma_{d}\left(1-\gamma_{d}\right){\left(1-\gamma_{E}\right)}^{2}}{{\left[1-\left(1-\gamma_{d}\right)\left(1-\gamma_{E}\right)\right]}^{2}}\right)}{p\left(1-\gamma_{E}\right)\left(1-\left(1-p\right)\left(1-\gamma_{d}\right)-\frac{p\gamma_{E}}{1-\left(1-\gamma_{d}\right)\left(1-\gamma_{E}\right)}\right)}M_{1}\\ \; & \;+\frac{\left(1-p\right)\gamma_{E}\left(1+\frac{p\left(1-\gamma_{E}\right)\left[1-{\left(1-\gamma_{d}\right)}^{2}\left(1-\gamma_{E}\right)\right]}{{\left[1-\left(1-\gamma_{d}\right)\left(1-\gamma_{E}\right)\right]}^{2}}\right)}{p\left(1-\gamma_{E}\right)\left(1-\left(1-p\right)\left(1-\gamma_{d}\right)-\frac{p\gamma_{E}}{1-\left(1-\gamma_{d}\right)\left(1-\gamma_{E}\right)}\right)}M_{2} \end{array}$$

*and*

(80) $$h_{2}^{\prime }\left(1\right)=\frac{\left(1-\gamma_{d}\right)\left(1+\frac{p\gamma_{d}\left(1-\gamma_{d}\right){\left(1-\gamma_{E}\right)}^{2}}{{\left[1-\left(1-\gamma_{d}\right)\left(1-\gamma_{E}\right)\right]}^{2}}\right)M_{1}+\left(1+\frac{p\left(1-\gamma_{E}\right)\left[1-{\left(1-\gamma_{d}\right)}^{2}\left(1-\gamma_{E}\right)\right]}{{\left[1-\left(1-\gamma_{d}\right)\left(1-\gamma_{E}\right)\right]}^{2}}\right)M_{2}}{1-\left(1-p\right)\left(1-\gamma_{d}\right)-\frac{p\gamma_{E}}{1-\left(1-\gamma_{d}\right)\left(1-\gamma_{E}\right)}}$$

**Proof.**  The proof of Theorem 4 is similar to that of Theorem 3. Thus, here we omit the derivation details of Theorem 4. □

## 5. Numerically Analyzing Relationships Between Freshness and Transmission Security

In Section 5.1, through numerical examples, we first investigate the change trends of average δ−secure AoI and insecure packet proportion with respect to deletion probability δ, as well as the tradeoffs between these two quantities. For the two cases discussed in Section 4, the average AoI with a random geometric service time deadline is numerically analyzed in Section 5.2. Letting δ=1, we depict the relation curves of the average AoSI versus packet generation probability *p*, and eavesdropping probability $\gamma_{E}$ in Section 5.3.

### 5.1. Average δ−Secure AoI and Insecure Packet Proportion

**Figure 2:**
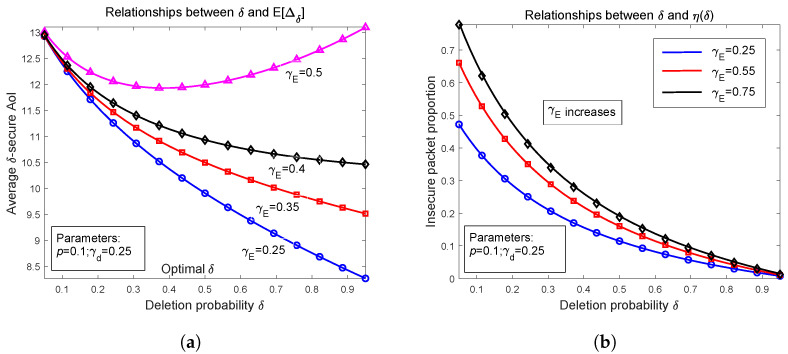
Numerical examples of average δ−secure AoI and insecure packet proportion versus deletion probability δ. (**a**) Relations between ${\bar{\Delta }}_{\delta }$ and probability δ; (**b**) Insecure packet proportion η(δ) vs. δ.

First of all, in Figure 2a we depict the relationship curves showing how ${\bar{\Delta }}_{\delta }$ varies when increasing packet deletion probability δ from 0 to 1. We select p=0.1, $\gamma_{d}=0.25$, and plot four curves by setting eavesdropping probability $\gamma_{E}$ to be 0.25, 0.35, 0.4, and 0.5. Numerical results show that in general, ${\bar{\Delta }}_{\delta }$ is monotonically decreasing as δ becomes large, except for cases where $\gamma_{E}$ is much larger than $\gamma_{d}$. These results are explained as follows. Notice that a large δ implies that it is more likely to delete an insecure packet, which will reduce the number of insecure packets arriving at the receiver. In some cases, deleting these insecure packets helps to improve the freshness of the obtained packets because the newer packet in the buffer can enter the server earlier and arrives at the receiver earlier. The curves with $\gamma_{E}=0.25$, 0.35, and 0.4 are exactly this situation. However, when increasing $\gamma_{E}$ further, the probability that a transmitted packet becomes insecure becomes high, and a large δ will result in many packet deletions before they can arrive at the receiver, which deteriorates the freshness of the obtained packets. Remember that in the proof of Theorem 2, the probability that a transmitted packet becomes insecure, i.e., $\mathrm{Pr}(E_{1})$, is equal to

(81) $$\mathrm{Pr}\left(E_{1}\right)=\frac{\gamma_{E}}{1-\left(1-\gamma_{d}\right)\left(1-\gamma_{E}\right)}$$

which is increasing when $\gamma_{E}$ becomes large. It is proved by the curve corresponding to $\gamma_{E}=0.5$ that the average δ−secure AoI first falls then rises when δ becomes large from 0 to 1. From the numerical results in Figure 2a, we can conclude that in general cases, especially when $\gamma_{d}>\gamma_{E}$, the average δ−secure AoI is decreasing as the packet deletion probability δ increases. In extreme situations where $\gamma_{E}$ is much larger than $\gamma_{d}$, as δ increases, ${\bar{\Delta }}_{\delta }$ first falls then rises, and there exists one optimal deletion probability that minimizes ${\bar{\Delta }}_{\delta }$ (approximately 0.35 in Figure 2a).

In Figure 2b, we depict the insecure packet proportion η(δ) when increasing δ from 0 to 1. It is easy to understand that η(δ) decreases as δ becomes large because the number of insecure packets that are transmitted to the receiver is reduced by increasing the packet deletion probability. We also plot three curves where $\gamma_{E}$ is set to be 0.25, 0.55, and 0.75. As explained in the previous paragraph, $\mathrm{Pr}(E_{1})$ increases when $\gamma_{E}$ becomes large. As a result, for large $\gamma_{E}$, more packets change to be insecure, and the same deletion probability will cause a higher insecure packet proportion. It is verified in Figure 2b that the black curve with $\gamma_{E}=0.75$ has the highest η(δ).

**Figure 3:**
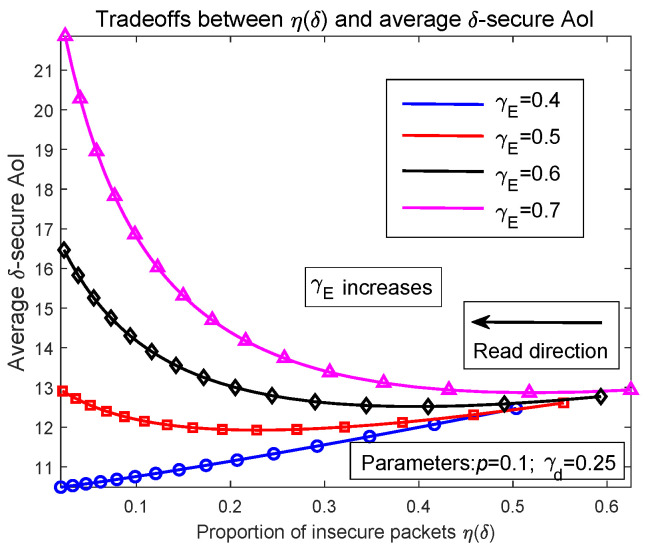
Tradeoffs between average δ−secure AoI and insecure packet proportion as a function of packet deletion probability δ.

By increasing packet deletion probability δ, we draw the relationship curves of ${\bar{\Delta }}_{\delta }$ and η(δ) in Figure 3 so as to investigate the direct tradeoffs of packet freshness and transmission security. Since η(δ) is decreasing when δ increases, Figure 3 is read from the right to the left. It is shown in Figure 3 that as $\gamma_{E}$ increases from 0.4 to 0.7, ${\bar{\Delta }}_{\delta }$ has different change trends when η(δ) varies. When $\gamma_{E}$ is 0.4, as η(δ) reduces, ${\bar{\Delta }}_{\delta }$ is decreasing as well. Increasing $\gamma_{E}$ to 0.5, ${\bar{\Delta }}_{\delta }$ first falls then rises. Finally, for extremely large $\gamma_{E}$, such as $\gamma_{E}=0.7$, ${\bar{\Delta }}_{\delta }$ is increasing when η(δ) reduces. These results have the same explanations as those for results in Figure 2. Extremely large $\gamma_{E}$ increases the probability that a packet becomes insecure. Then, when δ is large, there is a large probability that those insecure packet are deleted before being transmitted to the receiver, resulting in the average AoI increasing. Characterizing packet freshness by average AoI and representing transmission security using η(δ), numerical simulations in Figure 3 prove that depending on eavesdropping probability $\gamma_{E}$, ${\bar{\Delta }}_{\delta }$ varies in different trends with η(δ). In some cases, for example, the blue curve in Figure 3, both ${\bar{\Delta }}_{\delta }$ and η(δ) can be minimized by setting δ=1. However, in extreme cases where $\gamma_{E}$ is much larger than $\gamma_{d}$, when reducing η(δ), ${\bar{\Delta }}_{\delta }$ is increasing. That is, these two performance types cannot be optimized simultaneously.

**Figure 4:**
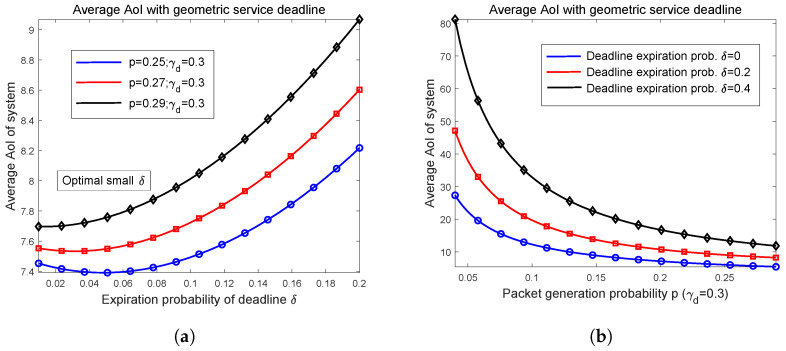
Relations between average AoI and δ, *p* in cases of system with random service time deadline. (**a**) Average AoI versus expiration probability; (**b**) Relations of average AoI and probability *p*.

Notice that in Figure 4 and Figure 7 of paper [5], the authors proved that the average AoI of the M/M/1/2 queue modeled system is decreasing when the packet arriving rate and service rate become large. If the probability of deleting a served packet is very small such that the average AoI is not significantly affected, in these cases, the average AoI of the system should have the similar change trends as in [5], that is, monotonically decreasing with *p* and $\gamma_{d}$. When $\gamma_{d}$ is greater than $\gamma_{E}$, according to Equation (81), the probability that a packet becomes insecure is very small such that even if δ equals 1, the probability of deleting a packet is still not very high. Consequently, the average AoI should follow a similar change trend to that in [5], which decreases with *p* and $\gamma_{d}$. On the other hand, increasing the packet deletion probability can always reduce insecure packet proportion, and in summary, we conclude that when $\gamma_{d}>\gamma_{E}$ such that the probability of deleting a packet is small, by setting δ=1, both the average AoI and η(δ) can be minimized at the same time. For extreme cases where $\gamma_{d}<\gamma_{E}$, i.e., the capability that the eavesdropper obtains a packet is stronger than the legitimate receiver, to obtain lower average δ−secure AoI, one should increase the emission power of the transmitter such that $\gamma_{d}$ is increased. If $\gamma_{E}$ is much larger than $\gamma_{d}$, then it is more appropriate to cancel the current transmission and restart sending the packet when $\gamma_{E}$ decreases.

### 5.2. Average AoI of the Ber/Geo/1/2 Queue Modeled System with Geometric Service Time Deadline

In this part, we provide numerical examples of average AoI for the Ber/Geo/1/2 queue modeled system when the packets are controlled by a random service time deadline. Firstly, the relationship curve between average AoI ${\bar{\Delta }}_{\delta }$ and expiration probability δ are illustrated in Figure 4a. By fixing $\gamma_{d}=0.3$ and increasing *p* from 0.25 to 0.29, we plot three curves. Referring also to Equation (81), when expiration probability δ is greater than $\gamma_{d}$, then many packets will be deleted before arriving at the receiver. This leads to an increase in the system’s average AoI. We limit the range of graphs in Figure 4a so as to make the varying trends prominent. The blue curve with p=0.25 shows that there exists an optimal but extremely small $\delta^{*}$ that minimizes ${\bar{\Delta }}_{\delta }$ (approximately 0.05 in Figure 4a), and for other two cases with larger *p*, ${\bar{\Delta }}_{\delta }$ is monotonically increasing as δ varies. Overall, the numerical results in Figure 4a prove that for most values of $\rho_{d}$, which is denoted as $p/\gamma_{d}$, letting δ=0, i.e., without using a random service time deadline can achieve the minimal average AoI. Only in some extreme cases where $\rho_{d}$ is very high can ${\bar{\Delta }}_{\delta }$ be slightly reduced by setting a very small expiration probability.

Remember that in work [6,7], the authors demonstrated that by imposing a carefully selected waiting time deadline, one can achieves lower average AoI for M/M/1/2 queue modeled and infinity capacity status updating systems. The waiting time deadline can reduce the average AoI, while in most cases the service time deadline cannot; this reveals that if you want to delete one packet for reducing average AoI, then it is better to delete it in the earlier stages.

We depict the relations between ${\bar{\Delta }}_{\delta }$ and *p* in Figure 4b, in which $\gamma_{d}=0.3$ and *p* varies from 0 to 0.29 such that $\rho_{d}$ takes the value from 0 to approximately 1. It is observed that for three cases of δ=0, 0.2, and 0.4, ${\bar{\Delta }}_{\delta }$ is decreasing with respect to *p*, as well as $\rho_{d}$. In addition, the curve corresponding to δ=0 is located at the bottom such that letting δ=0, or without using a random deadline in service process, achieves the minimal average AoI for the Ber/Geo/1/2 queue modeled system.

### 5.3. Average AoI of Secure Information for Ber/Geo/1/2 Queue Modeled System

**Figure 5:**
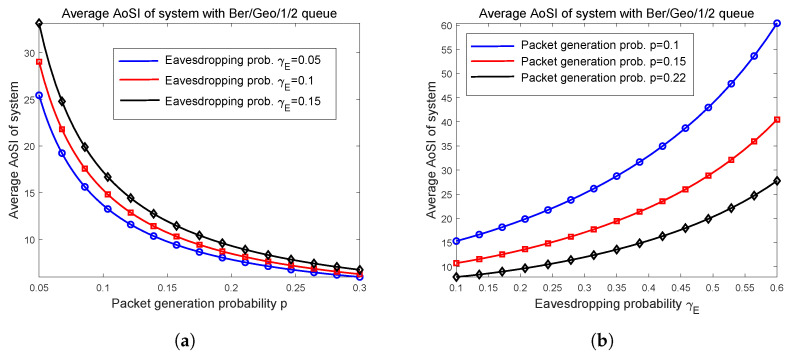
Average AoSI varies as packet generation probability *p* and eavesdropping probability $\gamma_{E}$ increases. (**a**) Average AoSI varies as *p* increases; (**b**) Relations between average AoSI and $\gamma_{E}$.

Finally, we investigate how the average AoSI varies when system parameters *p* and $\gamma_{E}$ change through numerical examples in Figure 5. It is shown in Figure 5a that the average AoSI ${\bar{\Delta }}_{S}$ decreases as *p* becomes large, and Figure 5b demonstrates that ${\bar{\Delta }}_{S}$ is increasing when the eavesdropping probability $\gamma_{E}$ varies from small to large. Here, the main observation is that the relationships between ${\bar{\Delta }}_{S}$ and *p*, and that between ${\bar{\Delta }}_{S}$ and $\gamma_{E}$, are both monotonic. Apart from the endpoints, there is no other $p^{*}$ or $\gamma_{E}^{*}$ that minimizes or maximizes ${\bar{\Delta }}_{S}$. Notice that ${\bar{\Delta }}_{S}$ characterizes the average age of the secure packet; thus, increasing the eavesdropping probability $\gamma_{E}$ will deteriorate ${\bar{\Delta }}_{S}$, which is shown in Figure 5a where black curve with $\gamma_{E}=0.15$ is located at the top, and is directly illustrated in Figure 5b. Again, this can be explained as $\mathrm{Pr}(E_{1})$, i.e., the probability that a packet changes to be insecure is increasing as $\gamma_{E}$ becomes large, which leads to more packet deletions before they can arrive at the receiver.

## 6. Discussion and Conclusions

Assuming that there is an eavesdropper, we consider transmitting packets in a timely manner to the receiver through a Ber/Geo/1/2 queue modeled status updating system. The aim is investigating the tradeoffs between information freshness and transmission security. In this paper, packet freshness is measured by the age of information, and we use the insecure packet proportion to represent system’s transmission security, where a packet is defined to be insecure if it is obtained by the eavesdropper earlier than the receiver. We derive the formula of the average AoI ${\bar{\Delta }}_{\delta }$, and the proportion of obtained insecure packets η(δ), under a probabilistic deletion/retaining transmission scheme. This enables us to analyze the relationships between these two performance types. As a special case of ${\bar{\Delta }}_{\delta }$, we determine the average AoI of the system in which the transmitted packets are limited by a random service time deadline, and the average age of secure AoI. Through numerical simulations, the main conclusion is that we demonstrate that the average AoI ${\bar{\Delta }}_{\delta }$ varies in different trends depending on the value of eavesdropping probability $\gamma_{E}$. In general cases when $\gamma_{d}>\gamma_{E}$, both ${\bar{\Delta }}_{\delta }$ and η(δ) are minimized by setting δ=1. While for extreme cases where $\gamma_{E}$ is very large, ${\bar{\Delta }}_{\delta }$ is increasing as η(δ) reduces such that ${\bar{\Delta }}_{\delta }$ and η(δ) cannot be optimized at the same time. For a system with other queuing models, for example, when the service time is deterministic, or assuming that the used deadline itself follows an arbitrary deadline, it is interesting to find the impacts of restricting the packet waiting time or service time on the average AoI of the system.

We discuss the tradeoffs of transmission timeliness and transmission security for infinity capacity system in work [41], in which we define freshness advantage *F* as a security measurement. In [41], *F* was defined as the average instantaneous gap between the eavesdropper’s AoI and legitimate receiver’s AoI. Here, it is interesting to compare the rationality of two transmission security measurements η(δ) and *F*. Optimizing *F* aims to increase the time interval between the legitimate receiver and the eavesdropper obtaining the same packet, while controlling η(δ) focuses on reducing the insecure packets obtained by the legitimate receiver and isolating the impact of the insecure packets on the system’s transmission security. From the authors’ point of view, if there are strong correlations between the transmitted packets, using *F* as a security measurement is more appropriate. Improving *F* expands the time interval between the legitimate receiver and eavesdropper obtaining the packets, which helps to reduce the possibility of the eavesdropper inferring other packets based on those he/she has obtained. However, for other cases of sending independent data, such as transmitting encoded packets, η(δ) is more suitable because reducing insecure packets and using secure packets to restore original data can effectively enhance the security of transmitted information.

Combining the two performance types derived in the paper, i.e., $\left({\bar{\Delta }}_{\delta },\eta \left(\delta \right)\right)$, we establish the following achievability relationships: we can achieve average AoI ${\bar{\Delta }}_{\delta }$ under the restriction that the proportion of obtained insecure packets is at most η(δ). Then, a natural question is what is the minimum average AoI that the receiver can obtain when the insecure packet proportion is no larger than a certain threshold? This is one of the follow-up problems that we will consider in the future. In addition, we desire to find the freshness advantage *F* for the Ber/Geo/1/2 queue modeled status updating system. By comparing with *F* of infinite capacity systems, the effects of the buffer size on the freshness advantage can be analyzed. Notice that the average AoIs of systems with the Ber/Geo/1/2 queue and Ber/Geo/1/∞ queue vary in different trends with traffic intensity, so it is expected that there are many interesting relations and tradeoffs to be analyzed. Apart from this, there is still much work in studying the relationship between transmission timeliness and transmission security. For example, introducing encryption and decryption operations in real-time packet transmission, and exploring the relationship between a system’s real-time performance and transmission security if the packets are encoded before being sent to the receiver.

## Figures and Tables

**Table 1:** State vector transfers of (n,m,l,0) and (n,m,l,1).

Initial State Vector	Considered Random Variables	State Vector at Next Time Slot
(n,m,l,0)n>m>l≥1	$\left(B_{d}=0,B_{E}=0\right)$	(n+1,m+1,l+1,0)
	$\left(B_{d}=0,B_{E}=1\right)$	F=0: (n+1,m+1,l+1,1)
		F=1: (n+1,l+1,0,0)
	$\left(B_{d}=1,B_{E}=0\right)$	(m+1,l+1,0,0)
	$\left(B_{d}=1,B_{E}=1\right)$	F=0: (m+1,l+1,0,0)
		F=1: (n+1,l+1,0,0)
(n,m,l,1)n>m>l≥1	F=0	$B_{d}=0$: (n+1,m+1,l+1,1)
		$B_{d}=1$: (m+1,l+1,0,0)
	F=1	(n+1,l+1,0,0)
(n,m,0,0)n>m≥1	$\left(A=0,B_{d}=0,B_{E}=0\right)$	(n+1,m+1,0,0)
	$\left(A=0,B_{d}=0,B_{E}=1\right)$	F=0: (n+1,m+1,0,1)
		F=1: (n+1,0,0,0)
	$\left(A=0,B_{d}=1,B_{E}=0\right)$	(m+1,0,0,0)
	$\left(A=0,B_{d}=1,B_{E}=1\right)$	F=0: (m+1,0,0,0)
		F=1: (n+1,0,0,0)
	$\left(A=1,B_{d}=0,B_{E}=0\right)$	(n+1,m+1,1,0)
	$\left(A=1,B_{d}=0,B_{E}=1\right)$	F=0: (n+1,m+1,1,1)
		F=1: (n+1,1,0,0)
	$\left(A=1,B_{d}=1,B_{E}=0\right)$	(m+1,1,0,0)
	$\left(A=1,B_{d}=1,B_{E}=1\right)$	F=0: (m+1,1,0,0)
		F=1: (n+1,1,0,0)
(n,m,0,1)n>m≥1	(A=0,F=0)	$B_{d}=0$: (n+1,m+1,0,1)
		$B_{d}=1$: (m+1,0,0,0)
	(A=0,F=1)	(n+1,0,0,0)
	(A=1,F=0)	$B_{d}=0$: (n+1,m+1,1,1)
		$B_{d}=1$: (m+1,1,0,0)
	(A=1,F=1)	(n+1,1,0,0)
(n,0,0,0)n≥1	A=0	(n+1,0,0,0)
	$\left(A=1,B_{d}=0,B_{E}=0\right)$	(n+1,1,0,0)
	$\left(A=1,B_{d}=0,B_{E}=1\right)$	F=0: (n+1,1,0,1)
		F=1: (n+1,0,0,0)
	$\left(A=1,B_{d}=1,B_{E}=0\right)$	(1,0,0,0)
	$\left(A=1,B_{d}=1,B_{E}=1\right)$	F=0: (1,0,0,0)
		F=1: (n+1,0,0,0)

## Data Availability

The original contributions presented in this study are included in the article. Further inquiries can be directed to the corresponding author.

## Appendix A. Proof of Lemma 1

We prove the relationships given in Lemma 1.

According to its definition and use stationary Equation (1), we have that

$$\begin{array}{cc} h_{3}\left(z\right) & =\sum_{m=1}^{\infty }\sum_{n=m+1}^{\infty }z^{n}\pi_{\left(n,m,0,1\right)}\\ \; & =\sum_{n=2}^{\infty }z^{n}\pi_{\left(n,1,0,1\right)}+\sum_{m=2}^{\infty }\sum_{n=m+1}^{\infty }z^{n}\pi_{\left(n,m,0,1\right)}\\ \; & =\sum_{n=2}^{\infty }z^{n}\pi_{\left(n-1,0,0,0\right)}p\left(1-\gamma_{d}\right)\gamma_{E}\left(1-\delta \right)\\ \; & \;+\sum_{m=2}^{\infty }\sum_{n=m+1}^{\infty }z^{n}\left(\pi_{\left(n-1,m-1,0,0\right)}\left(1-p\right)\left(1-\gamma_{d}\right)\gamma_{E}\left(1-\delta \right)+\pi_{\left(n-1,m-1,0,1\right)}\left(1-p\right)\left(1-\gamma_{d}\right)\left(1-\delta \right)\right)\\ \; & =p\left(1-\gamma_{d}\right)\gamma_{E}\left(1-\delta \right)zh_{1}\left(z\right)+\left(1-p\right)\left(1-\gamma_{d}\right)\gamma_{E}\left(1-\delta \right)zh_{2}\left(z\right)+\left(1-p\right)\left(1-\gamma_{d}\right)\left(1-\delta \right)zh_{3}\left(z\right) \end{array}$$

from which we obtain

(A1) $$\begin{array}{cc} \; & h_{3}\left(z\right)\left[1-\left(1-p\right)\left(1-\gamma_{d}\right)\left(1-\delta \right)z\right]\\ \; & \;=p\left(1-\gamma_{d}\right)\gamma_{E}\left(1-\delta \right)zh_{1}\left(z\right)+\left(1-p\right)\left(1-\gamma_{d}\right)\gamma_{E}\left(1-\delta \right)zh_{2}\left(z\right) \end{array}$$

For functions $h_{4}\left(z\right)$ and $h_{5}\left(z\right)$, firstly we derive the following two relations about probabilities $\pi_{\left(n,m,l,0\right)}$ and $\pi_{\left(n,m,l,1\right)}$.

From the first two rows of Equation (1), it is shown that

(A2) $$\pi_{\left(n,m,l,0\right)}=\pi_{\left(n-1,m-1,l-1,0\right)}\left(1-\gamma_{d}\right)\left(1-\gamma_{E}\right)=\ldots =\pi_{\left(n-l,m-l,0,0\right)}p{\left[\left(1-\gamma_{d}\right)\left(1-\gamma_{E}\right)\right]}^{l}$$

For probability $\pi_{\left(n,m,l,1\right)}$, the third row of Equation (1) shows that

(A3) $$\pi_{\left(n,m,l,1\right)}=\pi_{\left(n-1,m-1,l-1,1\right)}\left(1-\gamma_{d}\right)\left(1-\delta \right)+\pi_{\left(n-1,m-1,l-1,0\right)}\left(1-\gamma_{d}\right)\gamma_{E}\left(1-\delta \right)$$

which is the recursive relation of $\pi_{\left(n,m,l,1\right)}$. Applying (A3) repeatedly and using the fourth row of (1), one can verify that for n>m>l≥1,

(A4) $$\pi_{\left(n,m,l,1\right)}=\pi_{\left(n-l,m-l,0,0\right)}\frac{p\gamma_{E}\left(1-\delta \right)\left({\left[\left(1-\gamma_{d}\right)\left(1-\gamma_{E}\right)\right]}^{l}-{\left[\left(1-\gamma_{d}\right)\left(1-\delta \right)\right]}^{l}\right)}{\left(1-\gamma_{E}\right)-\left(1-\delta \right)}+\pi_{\left(n-l,m-l,0,1\right)}p{\left[\left(1-\gamma_{d}\right)\left(1-\delta \right)\right]}^{l}$$

Using (A2) and (A4), now $h_{4}\left(z\right)$ and $h_{5}\left(z\right)$ can be calculated:

(A5) $$\begin{array}{cc} h_{4}\left(z\right) & =\sum_{l=1}^{\infty }\sum_{m=l+1}^{\infty }\sum_{n=m+1}^{\infty }z^{n}\pi_{\left(n,m,l,0\right)}\\ \; & =\sum_{l=1}^{\infty }\sum_{m=l+1}^{\infty }\sum_{n=m+1}^{\infty }z^{n}\pi_{\left(n-l,m-l,0,0\right)}p{\left[\left(1-\gamma_{d}\right)\left(1-\gamma_{E}\right)\right]}^{l}\\ \; & =\sum_{l=1}^{\infty }\left(\sum_{\tilde{m}=1}^{\infty }\sum_{\tilde{n}=\tilde{m}+1}^{\infty }z^{\tilde{n}}\pi_{\left(\tilde{n},\tilde{m},0,0\right)}\right)p{\left[\left(1-\gamma_{d}\right)\left(1-\gamma_{E}\right)z\right]}^{l}\\ \; & =\frac{p\left(1-\gamma_{d}\right)\left(1-\gamma_{E}\right)z}{1-\left(1-\gamma_{d}\right)\left(1-\gamma_{E}\right)z}h_{2}\left(z\right) \end{array}$$

and

$$\begin{array}{cc} h_{5}\left(z\right) & =\sum_{l=1}^{\infty }\sum_{m=l+1}^{\infty }\sum_{n=m+1}^{\infty }z^{n}\pi_{\left(n,m,l,1\right)}\\ \; & =\sum_{l=1}^{\infty }\left(\sum_{m=l+1}^{\infty }\sum_{n=m+1}^{\infty }z^{n}\pi_{\left(n-l,m-l,0,0\right)}\right)\frac{p\gamma_{E}\left(1-\delta \right)\left({\left[\left(1-\gamma_{d}\right)\left(1-\gamma_{E}\right)\right]}^{l}-{\left[\left(1-\gamma_{d}\right)\left(1-\delta \right)\right]}^{l}\right)}{\left(1-\gamma_{E}\right)-\left(1-\delta \right)}\\ \; & \;+\sum_{l=1}^{\infty }\left(\sum_{m=l+1}^{\infty }\sum_{n=m+1}^{\infty }z^{n}\pi_{\left(n-l,m-l,0,1\right)}\right)p{\left[\left(1-\gamma_{d}\right)\left(1-\delta \right)\right]}^{l} \end{array}$$

(A6) $$\begin{array}{cc} \; & \;=h_{2}\left(z\right)\sum_{l=1}^{\infty }\frac{p\gamma_{E}\left(1-\delta \right)\left({\left[\left(1-\gamma_{d}\right)\left(1-\gamma_{E}\right)z\right]}^{l}-{\left[\left(1-\gamma_{d}\right)\left(1-\delta \right)z\right]}^{l}\right)}{\left(1-\gamma_{E}\right)-\left(1-\delta \right)}+h_{3}\left(z\right)\sum_{l=1}^{\infty }p{\left[\left(1-\gamma_{d}\right)\left(1-\delta \right)z\right]}^{l}\\ \; & \;=\frac{p\gamma_{E}\left(1-\gamma_{d}\right)\left(1-\delta \right)z}{\left[1-\left(1-\gamma_{d}\right)\left(1-\gamma_{E}\right)z\right]\left[1-\left(1-\gamma_{d}\right)\left(1-\delta \right)z\right]}h_{2}\left(z\right)+\frac{p\left(1-\gamma_{d}\right)\left(1-\delta \right)z}{1-\left(1-\gamma_{d}\right)\left(1-\delta \right)z}h_{3}\left(z\right) \end{array}$$

Collecting Equations (A1), (A5) and (A6), the proof of Lemma 1 is completed.

## Appendix B. Proof of Lemma 2

In this appendix, we derive the results given in Lemma 2.

Using Equation (1), we first calculate $h_{1}\left(z\right)$. According to its definition and (1),

(A7) $$\begin{array}{cc} h_{1}\left(z\right) & =\sum_{n=1}^{\infty }z^{n}\pi_{\left(n,0,0,0\right)}\\ \; & =z\pi_{\left(1,0,0,0\right)}+\sum_{n=2}^{\infty }z^{n}\pi_{\left(n,0,0,0\right)}\\ \; & =z\left(\sum_{k=1}^{\infty }\pi_{\left(k,0,0,0\right)}\right)p\gamma_{d}\left(1-\gamma_{E}\delta \right)\\ \; & \;+\sum_{n=2}^{\infty }z^{n}[\pi_{\left(n-1,0,0,0\right)}\left[\left(1-p\right)+p\gamma_{E}\delta \right]+\sum_{k=n}^{\infty }\pi_{\left(k,n-1,0,0\right)}\left(1-p\right)\gamma_{d}\left(1-\gamma_{E}\delta \right)\\ \; & \;+\sum_{j=1}^{n-2}\pi_{\left(n-1,j,0,0\right)}\left(1-p\right)\gamma_{E}\delta +\sum_{k=n}^{\infty }\pi_{\left(k,n-1,0,1\right)}\left(1-p\right)\gamma_{d}\left(1-\delta \right)\\ \; & \;+\sum_{j=1}^{n-2}\pi_{\left(n-1,j,0,1\right)}\left(1-p\right)\delta ]\\ \; & =p\gamma_{d}\left(1-\gamma_{E}\delta \right)M_{1}z+\left[\left(1-p\right)+p\gamma_{E}\delta \right]zh_{1}\left(z\right)+\left(1-p\right)\gamma_{d}\left(1-\gamma_{E}\delta \right)zh_{2}^{\left(m\right)}\left(z\right)\\ \; & \;+\left(1-p\right)\gamma_{E}\delta zh_{2}\left(z\right)+\left(1-p\right)\gamma_{d}\left(1-\delta \right)zh_{3}^{\left(m\right)}\left(z\right)+\left(1-p\right)\delta zh_{3}\left(z\right) \end{array}$$

in which we define

(A8) $$h_{2}^{\left(m\right)}\left(z\right)=\sum_{m=1}^{\infty }\sum_{n=m+1}^{\infty }z^{m}\pi_{\left(n,m,0,0\right)},\;h_{3}^{\left(m\right)}\left(z\right)=\sum_{m=1}^{\infty }\sum_{n=m+1}^{\infty }z^{m}\pi_{\left(n,m,0,1\right)}$$

Notice that in obtaining $h_{2}^{\left(m\right)}\left(z\right)$ and $h_{3}^{\left(m\right)}\left(z\right)$ in (A8), we have replaced the variables in the original summations.

Similarly, for function $h_{2}\left(z\right)$ we have that

$$\begin{array}{cc} h_{2}\left(z\right) & =\sum_{m=1}^{\infty }\sum_{n=m+1}^{\infty }z^{n}\pi_{\left(n,m,0,0\right)}=\sum_{n=2}^{\infty }z^{n}\pi_{\left(n,1,0,0\right)}+\sum_{m=2}^{\infty }\sum_{n=m+1}^{\infty }z^{n}\pi_{\left(n,m,0,0\right)} \end{array}$$

Using the equations in (1) and calculating each summation term, it is shown that

(A9) $$\begin{array}{cc} h_{2}\left(z\right) & =p\left(1-\gamma_{d}\right)\left(1-\gamma_{E}\right)zh_{1}\left(z\right)+p\gamma_{d}\left(1-\gamma_{E}\delta \right)zh_{2}^{\left(m\right)}\left(z\right)+p\gamma_{E}\delta zh_{2}\left(z\right)\\ \; & \;+p\gamma_{d}\left(1-\delta \right)zh_{3}^{\left(m\right)}\left(z\right)+p\delta zh_{3}\left(z\right)+\left(1-p\right)\left(1-\gamma_{d}\right)\left(1-\gamma_{E}\right)zh_{2}\left(z\right)\\ \; & \;+\gamma_{d}\left(1-\gamma_{E}\delta \right)zh_{4}^{\left(m\right)}\left(z\right)+\gamma_{E}\delta zh_{4}\left(z\right)+\gamma_{d}\left(1-\delta \right)zh_{5}^{\left(m\right)}\left(z\right)+\delta zh_{5}\left(z\right) \end{array}$$

where

(A10) $$\begin{array}{c} h_{4}^{\left(m\right)}\left(z\right)=\sum_{l=1}^{\infty }\sum_{m=l+1}^{\infty }\sum_{n=m+1}^{\infty }z^{m}\pi_{\left(n,m,l,0\right)} \end{array}$$

(A11) $$\begin{array}{c} h_{5}^{\left(m\right)}\left(z\right)=\sum_{l=1}^{\infty }\sum_{m=l+1}^{\infty }\sum_{n=m+1}^{\infty }z^{m}\pi_{\left(n,m,l,1\right)} \end{array}$$

To simplify Equations (A7) and (A9), we eliminate $h_{3}\left(z\right)$, $h_{4}\left(z\right)$, and $h_{5}\left(z\right)$ using Lemma 1. In addition, we show without detailed calculations that the relationships given in Lemma 1 also hold for functions $h_{i}^{\left(m\right)}\left(z\right)$, 2≤i≤5. More specifically, we prove that

(A12) $$\begin{array}{c} h_{3}^{\left(m\right)}\left(z\right)=\frac{p\left(1-\gamma_{d}\right)\gamma_{E}\left(1-\delta \right)M_{1}z}{1-\left(1-p\right)\left(1-\gamma_{d}\right)\left(1-\delta \right)z}+\frac{\left(1-p\right)\left(1-\gamma_{d}\right)\gamma_{E}\left(1-\delta \right)z}{1-\left(1-p\right)\left(1-\gamma_{d}\right)\left(1-\delta \right)z}h_{2}^{\left(m\right)}\left(z\right) \end{array}$$

(A13) $$\begin{array}{c} h_{4}^{\left(m\right)}\left(z\right)=\frac{p\left(1-\gamma_{d}\right)\left(1-\gamma_{E}\right)z}{1-\left(1-\gamma_{d}\right)\left(1-\gamma_{E}\right)z}h_{2}^{\left(m\right)}\left(z\right) \end{array}$$

(A14) $$\begin{array}{c} h_{5}^{\left(m\right)}\left(z\right)=\frac{p\left(1-\gamma_{d}\right)\gamma_{E}\left(1-\delta \right)z}{\left[1-\left(1-\gamma_{d}\right)\left(1-\gamma_{E}\right)z\right]\left[1-\left(1-\gamma_{d}\right)\left(1-\delta \right)z\right]}h_{2}^{\left(m\right)}\left(z\right)+\frac{p\left(1-\gamma_{d}\right)\left(1-\delta \right)z}{1-\left(1-\gamma_{d}\right)\left(1-\delta \right)z}h_{3}^{\left(m\right)}\left(z\right) \end{array}$$

Applying (A12)–(A14), $h_{3}^{\left(m\right)}\left(z\right)$, $h_{4}^{\left(m\right)}\left(z\right)$, and $h_{5}^{\left(m\right)}\left(z\right)$ in (A7) and (A9) can be represented by $M_{1}$ and $h_{2}^{\left(m\right)}\left(z\right)$.

Omitting the calculation details, we prove that (A7) and (A9) are equivalent to

(A15) $$\begin{array}{cc} \; & h_{1}\left(z\right)\left(1-\left(1-p\right)z-\frac{p\gamma_{E}\delta z}{1-\left(1-p\right)\left(1-\gamma_{d}\right)\left(1-\delta \right)z}\right)\\ \; & \;=\frac{\left(1-p\right)\gamma_{E}\delta z}{1-\left(1-p\right)\left(1-\gamma_{d}\right)\left(1-\delta \right)z}h_{2}\left(z\right)+p\gamma_{d}\left(1-\gamma_{E}\right)M_{1}z+\frac{p\gamma_{d}\gamma_{E}\left(1-\delta \right)M_{1}z}{1-\left(1-p\right)\left(1-\gamma_{d}\right)\left(1-\delta \right)z}\\ \; & \;+\left(\left(1-p\right)\gamma_{d}\left(1-\gamma_{E}\right)z+\frac{\left(1-p\right)\gamma_{d}\gamma_{E}\left(1-\delta \right)z}{1-\left(1-p\right)\left(1-\gamma_{d}\right)\left(1-\delta \right)z}\right)h_{2}^{\left(m\right)}\left(z\right) \end{array}$$

and

(A16) $$\begin{array}{cc} \; & h_{2}\left(z\right)\left[1-\left(1-p\right)\left(1-\gamma_{d}\right)\left(1-\gamma_{E}\right)z-\frac{p\gamma_{E}\delta z\left(\frac{1}{1-\left(1-\gamma_{d}\right)\left(1-\gamma_{E}\right)z}+\frac{\left(1-p\right)\left(1-\gamma_{d}\right)\left(1-\delta \right)z}{1-\left(1-p\right)\left(1-\gamma_{d}\right)\left(1-\delta \right)z}\right)}{1-\left(1-\gamma_{d}\right)\left(1-\delta \right)z}\right]\\ \; & \;=h_{1}\left(z\right)\left(p\left(1-\gamma_{d}\right)\left(1-\gamma_{E}\right)z+\frac{p^{2}\gamma_{E}\delta \left(1-\gamma_{d}\right)\left(1-\delta \right)z^{2}}{\left[1-\left(1-\gamma_{d}\right)\left(1-\delta \right)z\right]\left[1-\left(1-p\right)\left(1-\gamma_{d}\right)\left(1-\delta \right)z\right]}\right)\\ \; & \;+h_{2}^{\left(m\right)}\left(z\right)\left(\frac{p\gamma_{d}z\left[1-\gamma_{E}\delta -\left(1-\gamma_{d}\right)\left(1-\gamma_{E}\right)\left(1-\delta \right)z\right]}{\left[1-\left(1-\gamma_{d}\right)\left(1-\gamma_{E}\right)z\right]\left[1-\left(1-\gamma_{d}\right)\left(1-\delta \right)z\right]}+\frac{p\gamma_{d}\gamma_{E}\left(1-p\right)\left(1-\gamma_{d}\right){\left(1-\delta \right)}^{2}z^{2}}{\left[1-\left(1-\gamma_{d}\right)\left(1-\delta \right)z\right]\left[1-\left(1-p\right)\left(1-\gamma_{d}\right)\left(1-\delta \right)z\right]}\right)\\ \; & \;+\frac{p^{2}\gamma_{d}\gamma_{E}\left(1-\gamma_{d}\right){\left(1-\delta \right)}^{2}M_{1}z^{2}}{\left[1-\left(1-\gamma_{d}\right)\left(1-\delta \right)z\right]\left[1-\left(1-p\right)\left(1-\gamma_{d}\right)\left(1-\delta \right)z\right]} \end{array}$$

Calculating the derivative of *z* from both sides of (A15) and (A16), omitting those direct calculations, we obtain that

(A17) $$\begin{array}{cc} \; & p\left(1-\frac{\gamma_{E}\delta }{1-\left(1-p\right)\left(1-\gamma_{d}\right)\left(1-\delta \right)}\right)h_{1}^{\prime }\left(1\right)-\frac{\left(1-p\right)\gamma_{E}\delta }{1-\left(1-p\right)\left(1-\gamma_{d}\right)\left(1-\delta \right)}h_{2}^{\prime }\left(1\right)\\ = & \left(\left(1-p\right)+p\gamma_{d}\left(1-\gamma_{E}\right)+\frac{p\gamma_{E}\left[1-\left(1-\gamma_{d}\right)\left(1-\delta \right)\right]}{{\left[1-\left(1-p\right)\left(1-\gamma_{d}\right)\left(1-\delta \right)\right]}^{2}}\right)M_{1}\\ & +\left(\left(1-p\right)\gamma_{d}\left(1-\gamma_{E}\right)+\frac{\left(1-p\right)\gamma_{E}\left[1-\left(1-\gamma_{d}\right)\left(1-\delta \right)\right]}{{\left[1-\left(1-p\right)\left(1-\gamma_{d}\right)\left(1-\delta \right)\right]}^{2}}\right)M_{2}\\ & +\left(\left(1-p\right)\gamma_{d}\left(1-\gamma_{E}\right)+\frac{\left(1-p\right)\gamma_{d}\gamma_{E}\left(1-\delta \right)}{1-\left(1-p\right)\left(1-\gamma_{d}\right)\left(1-\delta \right)}\right)\left[{\left.\frac{dh_{2}^{\left(m\right)}\left(z\right)}{dz}\right|}_{z=1}\right] \end{array}$$

and

(A18) $$\begin{array}{cc} \; & \left[-p\left(1-\gamma_{d}\right)\left(1-\gamma_{E}\right)+p\gamma_{E}\delta \left(\frac{1}{1-\left(1-p\right)\left(1-\gamma_{d}\right)\left(1-\delta \right)}-\frac{1}{1-\left(1-\gamma_{d}\right)\left(1-\delta \right)}\right)\right]h_{1}^{\prime }\left(1\right)\\ \; & \;+\left[1-\left(1-p\right)\left(1-\gamma_{d}\right)\left(1-\gamma_{E}\right)-\frac{p\gamma_{E}\delta }{1-\left(1-\gamma_{d}\right)\left(1-\delta \right)}\left(\frac{1}{1-\left(1-\gamma_{d}\right)\left(1-\gamma_{E}\right)}+\frac{\left(1-p\right)\left(1-\gamma_{d}\right)\left(1-\gamma_{E}\right)}{1-\left(1-p\right)\left(1-\gamma_{d}\right)\left(1-\gamma_{E}\right)}\right)\right]h_{2}^{\prime }\left(1\right)\\ \; & =\left[p\left(1-\gamma_{d}\right)\left(1-\gamma_{E}\right)+p\gamma_{E}\left(\frac{1}{1-\left(1-\gamma_{d}\right)\left(1-\delta \right)}-\frac{1-\left(1-\gamma_{d}\right)\left(1-\delta \right)}{{\left[1-\left(1-p\right)\left(1-\gamma_{d}\right)\left(1-\delta \right)\right]}^{2}}\right)\right]M_{1}\\ \; & \;+[\left(1-p\right)\left(1-\gamma_{d}\right)\left(1-\gamma_{E}\right)+\frac{p[1-\left(1-p\right)\left(1-\gamma_{d}\right)\left(1-\delta \right)]}{\left[1-\left(1-\gamma_{d}\right)\left(1-\gamma_{E}\right)\right]\left[1-\left(1-\gamma_{d}\right)\left(1-\delta \right)\right]}\\ \; & \;+\frac{p\gamma_{E}\left(1-p\right)\left(1-\gamma_{d}\right)\left(1-\delta \right)}{1-\left(1-p\right)\left(1-\gamma_{d}\right)\left(1-\delta \right)}\left(\frac{1}{1-\left(1-\gamma_{d}\right)\left(1-\delta \right)}+\frac{1}{1-\left(1-p\right)\left(1-\gamma_{d}\right)\left(1-\delta \right)}\right)]M_{2}\\ \; & \;+\left[\frac{p\gamma_{d}\left[1-\gamma_{E}\delta -\left(1-\gamma_{d}\right)\left(1-\gamma_{E}\right)\left(1-\delta \right)\right]}{\left[1-\left(1-\gamma_{d}\right)\left(1-\gamma_{E}\right)\right]\left[1-\left(1-\gamma_{d}\right)\left(1-\delta \right)\right]}+\frac{p\gamma_{d}\gamma_{E}\left(1-p\right)\left(1-\gamma_{d}\right){\left(1-\delta \right)}^{2}}{\left[1-\left(1-\gamma_{d}\right)\left(1-\delta \right)\right]\left[1-\left(1-p\right)\left(1-\gamma_{d}\right)\left(1-\delta \right)\right]}\right]\left[{\left.\frac{dh_{2}^{\left(m\right)}\left(z\right)}{dz}\right|}_{z=1}\right] \end{array}$$

and this derives result (15) in Lemma 2.

Next, we determine $h_{2}^{\left(m\right)}\left(z\right)$, obtaining its derivative at z=1. Following the same method of calculating $h_{2}\left(z\right)$, we have the following equations.

Using stationary Equation (1),

$$\begin{array}{cc} h_{2}^{\left(m\right)}\left(z\right) & =\sum_{m=1}^{\infty }\sum_{n=m+1}^{\infty }z^{m}\pi_{\left(n,m,0,0\right)}\\ \; & =z\sum_{n=2}^{\infty }\pi_{\left(n,1,0,0\right)}+\sum_{m=2}^{\infty }\sum_{n=m+1}^{\infty }z^{m}\pi_{\left(n,m,0,0\right)}\\ \; & =z\sum_{n=2}^{\infty }(\pi_{\left(n-1,0,0,0\right)}p\left(1-\gamma_{d}\right)\left(1-\gamma_{E}\right)+\sum_{k=n}^{\infty }\pi_{\left(k,n-1,0,0\right)}p\gamma_{d}\left(1-\gamma_{E}\delta \right)+\sum_{j=1}^{n-2}\pi_{\left(n-1,j,0,0\right)}p\gamma_{E}\delta \\ \; & \;+\sum_{k=n}^{\infty }\pi_{\left(k,n-1,0,1\right)}p\gamma_{d}\left(1-\delta \right)+\sum_{j=1}^{n-2}\pi_{\left(n-1,j,0,1\right)}p\delta )\\ \; & \;+\sum_{m=2}^{\infty }\sum_{n=m+1}^{\infty }z^{m}(\pi_{\left(n-1,m-1,0,0\right)}\left(1-p\right)\left(1-\gamma_{d}\right)\left(1-\gamma_{E}\right)+\sum_{k=n}^{\infty }\pi_{\left(k,n-1,m-1,0\right)}\gamma_{d}\left(1-\gamma_{E}\delta \right)\\ \; & \;+\sum_{j=m}^{n-2}\pi_{\left(n-1,j,m-1,0\right)}\gamma_{E}\delta +\sum_{k=n}^{\infty }\pi_{\left(k,n-1,m-1,1\right)}\gamma_{d}\left(1-\delta \right)+\sum_{j=m}^{n-2}\pi_{\left(n-1,j,m-1,1\right)}\delta ) \end{array}$$

which is calculated to be

$$\begin{array}{cc} \; & p\left(1-\gamma_{d}\right)\left(1-\gamma_{E}\right)M_{1}z+p\gamma_{d}\left(1-\gamma_{E}\delta \right)M_{2}z+p\gamma_{E}\delta M_{2}z+p\gamma_{d}\left(1-\delta \right)M_{3}z+p\delta M_{3}z\\ \; & \;+\left(1-p\right)\left(1-\gamma_{d}\right)\left(1-\gamma_{E}\right)zh_{2}^{\left(m\right)}\left(z\right)+\left(\sum_{m=2}^{\infty }\sum_{n=m+1}^{\infty }z^{m}\sum_{k=n}^{\infty }\pi_{\left(k,n-1,m-1,0\right)}\right)\gamma_{d}\left(1-\gamma_{E}\delta \right)\\ \; & \;+\left(\sum_{m=2}^{\infty }\sum_{n=m+2}^{\infty }z^{m}\sum_{j=m}^{n-2}\pi_{\left(n-1,j,m-1,0\right)}\right)\gamma_{E}\delta \\ \; & \;+\left(\sum_{m=2}^{\infty }\sum_{n=m+1}^{\infty }z^{m}\sum_{k=n}^{\infty }\pi_{\left(k,n-1,m-1,1\right)}\right)\gamma_{d}\left(1-\delta \right)\\ \; & \;+\left(\sum_{m=2}^{\infty }\sum_{n=m+2}^{\infty }z^{m}\sum_{j=m}^{n-2}\pi_{\left(n-1,j,m-1,1\right)}\right)\delta \end{array}$$

By changing the variables and summation order, we show that

(A19) $$\begin{array}{cc} \; & \sum_{m=2}^{\infty }\sum_{n=m+1}^{\infty }z^{m}\sum_{k=n}^{\infty }\pi_{\left(k,n-1,m-1,0\right)}=\sum_{m=2}^{\infty }\sum_{n=m+2}^{\infty }z^{m}\sum_{j=m}^{n-2}\pi_{\left(n-1,j,m-1,0\right)}\\ = & \sum_{l^{\prime }=1}^{\infty }\sum_{m^{\prime }=l^{\prime }+1}^{\infty }\sum_{n^{\prime }=m^{\prime }+1}^{\infty }z^{l^{\prime }+1}\pi_{\left(n^{\prime },m^{\prime },l^{\prime },0\right)} \end{array}$$

and

(A20) $$\begin{array}{cc} \; & \sum_{m=2}^{\infty }\sum_{n=m+1}^{\infty }z^{m}\sum_{k=n}^{\infty }\pi_{\left(k,n-1,m-1,1\right)}=\sum_{m=2}^{\infty }\sum_{n=m+2}^{\infty }z^{m}\sum_{j=m}^{n-2}\pi_{\left(n-1,j,m-1,1\right)}\\ = & \sum_{l^{\prime }=1}^{\infty }\sum_{m^{\prime }=l^{\prime }+1}^{\infty }\sum_{n^{\prime }=m^{\prime }+1}^{\infty }z^{l^{\prime }+1}\pi_{\left(n^{\prime },m^{\prime },l^{\prime },1\right)} \end{array}$$

Applying Equations (A2) and (A3) in the proof of Lemma 1, it is calculated that

(A21) $$\sum_{l^{\prime }=1}^{\infty }\sum_{m^{\prime }=l^{\prime }+1}^{\infty }\sum_{n^{\prime }=m^{\prime }+1}^{\infty }z^{l^{\prime }+1}\pi_{\left(n^{\prime },m^{\prime },l^{\prime },0\right)}=\frac{p\left(1-\gamma_{d}\right)\left(1-\gamma_{E}\right)M_{2}z^{2}}{1-\left(1-\gamma_{d}\right)\left(1-\gamma_{E}\right)z}$$

and the second sum is equal to

(A22) $$\begin{array}{cc} \; & \sum_{l^{\prime }=1}^{\infty }\sum_{m^{\prime }=l^{\prime }+1}^{\infty }\sum_{n^{\prime }=m^{\prime }+1}^{\infty }z^{l^{\prime }+1}\pi_{\left(n^{\prime },m^{\prime },l^{\prime },0\right)}\\ = & \frac{p\gamma_{E}\left(1-\delta \right)M_{2}z}{\left(1-\gamma_{E}\right)-\left(1-\delta \right)}\left(\frac{\left(1-\gamma_{d}\right)\left(1-\gamma_{E}\right)z}{1-\left(1-\gamma_{d}\right)\left(1-\gamma_{E}\right)z}-\frac{\left(1-\gamma_{d}\right)\left(1-\delta \right)z}{1-\left(1-\gamma_{d}\right)\left(1-\delta \right)z}\right)\\ & +\frac{p\left(1-\gamma_{d}\right)\left(1-\delta \right)M_{3}z^{2}}{1-\left(1-\gamma_{d}\right)\left(1-\delta \right)z} \end{array}$$

Substituting (A21) and (A22), after some extra calculations, it is shown that

(A23) $$\begin{array}{cc} \; & h_{2}^{\left(m\right)}\left(z\right)\left[1-\left(1-p\right)\left(1-\gamma_{d}\right)\left(1-\gamma_{E}\right)z\right]=p\left(1-\gamma_{d}\right)\left(1-\gamma_{E}\right)M_{1}z+\frac{p\left[1-\left(1-\gamma_{d}\right)\left(1-\gamma_{E}\right)\right]M_{2}z}{1-\left(1-\gamma_{d}\right)\left(1-\gamma_{E}\right)z}\\ \; & \;\;+\frac{p\left[1-\left(1-\gamma_{d}\right)\left(1-\delta \right)\right]M_{3}z}{1-\left(1-\gamma_{d}\right)\left(1-\delta \right)z}-\frac{p\left(1-\gamma_{d}\right)\gamma_{E}\left(1-\delta \right)M_{2}z\left(1-z\right)}{\left[1-\left(1-\gamma_{d}\right)\left(1-\gamma_{E}\right)z\right]\left[1-\left(1-\gamma_{d}\right)\left(1-\delta \right)z\right]} \end{array}$$

Taking the derivative from both sides of (A23) and letting z=1, omitting tedious calculations, we give that

(A24) $$\begin{array}{cc} {\left.\frac{dh_{2}^{\left(m\right)}\left(z\right)}{dz}\right|}_{z=1} & =\frac{p\left(1-\gamma_{d}\right)\left(1-\gamma_{E}\right)+\frac{p^{2}\left(1-\gamma_{d}\right)\gamma_{E}\left(1-\delta \right)}{\left[1-\left(1-\gamma_{d}\right)\left(1-\delta \right)\right]\left[1-\left(1-p\right)\left(1-\gamma_{d}\right)\left(1-\delta \right)\right]}}{1-\left(1-p\right)\left(1-\gamma_{d}\right)\left(1-\gamma_{E}\right)}M_{1}\\ \; & +\frac{\left(1-p\right)\left(1-\gamma_{d}\right)\left(1-\gamma_{E}\right)+\frac{p[1-\left(1-\gamma_{d}\right)\left(1-\gamma_{E}\right)\left(1-\delta \right)]}{\left[1-\left(1-\gamma_{d}\right)\left(1-\gamma_{E}\right)\right]\left[1-\left(1-\gamma_{d}\right)\left(1-\delta \right)\right]}+\frac{p\left(1-p\right)\left(1-\gamma_{d}\right)\gamma_{E}\left(1-\delta \right)}{\left[1-\left(1-\gamma_{d}\right)\left(1-\delta \right)\right]\left[1-\left(1-p\right)\left(1-\gamma_{d}\right)\left(1-\delta \right)\right]}}{1-\left(1-p\right)\left(1-\gamma_{d}\right)\left(1-\gamma_{E}\right)}M_{2} \end{array}$$

This proves result (25) in Lemma 2.

Finally, we determine $M_{1}$ and $M_{2}$. From the definition of $H_{\delta }\left(z\right)$ in (4) and Equation (13), we have

(A25) $$\begin{array}{cc} 1 & =\left(1+\frac{p\left(1-\gamma_{d}\right)\gamma_{E}\left(1-\delta \right)z}{1-\left(1-\gamma_{d}\right)\left(1-\delta \right)z}\right)M_{1}\\ \; & \;+\frac{\left[1-\left(1-p\right)\left(1-\gamma_{d}\right)\left(1-\gamma_{E}\right)z\right]\left[1-\left(1-\gamma_{d}\right)\left(1-\gamma_{E}\right)\left(1-\delta \right)z\right]}{\left[1-\left(1-\gamma_{d}\right)\left(1-\gamma_{E}\right)z\right]\left[1-\left(1-\gamma_{d}\right)\left(1-\delta \right)z\right]}M_{2} \end{array}$$

Another relation between $M_{1}$ and $M_{2}$ is determined as follows.

Letting z=1 in Equation (A7) yields

(A26) $$p\left(1-\gamma_{d}\right)\left(1-\gamma_{E}\delta \right)M_{1}=\left(1-p\right)\left[1-\left(1-\gamma_{d}\right)\left(1-\gamma_{E}\delta \right)\right]M_{2}+\left(1-p\right)\left[1-\left(1-\gamma_{d}\right)\left(1-\delta \right)\right]M_{3}$$

while substituting z=1 into (10) gives

(A27) $$M_{3}=\frac{p\left(1-\gamma_{d}\right)\gamma_{E}\left(1-\delta \right)}{1-\left(1-p\right)\left(1-\gamma_{d}\right)\left(1-\delta \right)}M_{1}+\frac{\left(1-p\right)\left(1-\gamma_{d}\right)\gamma_{E}\left(1-\delta \right)}{1-\left(1-p\right)\left(1-\gamma_{d}\right)\left(1-\delta \right)}M_{2}$$

Notice that this relation is used in (A24) to eliminate $M_{3}$. Combining (A26) and (A27), we obtain that

(A28) $$\begin{array}{cc} M_{2}\; & =\frac{p\left(1-\gamma_{d}\right)\left(\left(1-\gamma_{E}\right)\left[1-\left(1-p\right)\left(1-\gamma_{d}\right)\left(1-\delta \right)\right]+p\gamma_{E}\left(1-\delta \right)\right)}{\left(1-p\right)\left(\left[1-\left(1-\gamma_{d}\right)\left(1-\gamma_{E}\right)\right]\left[1-\left(1-\gamma_{d}\right)\left(1-\delta \right)\right]+p\gamma_{d}\left(1-\gamma_{d}\right)\left(1-\gamma_{E}\right)\left(1-\delta \right)\right)}M_{1} \end{array}$$

Denote

(A29) $$R_{1,2}=\frac{p\left(1-\gamma_{d}\right)\left(\left(1-\gamma_{E}\right)\left[1-\left(1-p\right)\left(1-\gamma_{d}\right)\left(1-\delta \right)\right]+p\gamma_{E}\left(1-\delta \right)\right)}{\left(1-p\right)\left(\left[1-\left(1-\gamma_{d}\right)\left(1-\gamma_{E}\right)\right]\left[1-\left(1-\gamma_{d}\right)\left(1-\delta \right)\right]+p\gamma_{d}\left(1-\gamma_{d}\right)\left(1-\gamma_{E}\right)\left(1-\delta \right)\right)}$$

then, $M_{2}=R_{1,2}M_{1}$. Combining with (A25), we finally determine that

(A30) $$M_{1}={\left(1+\frac{p\left(1-\gamma_{d}\right)\gamma_{E}\left(1-\delta \right)}{1-\left(1-\gamma_{d}\right)\left(1-\delta \right)}+\frac{\left[1-\left(1-p\right)\left(1-\gamma_{d}\right)\left(1-\gamma_{E}\right)\right]\left[1-\left(1-\gamma_{d}\right)\left(1-\gamma_{E}\right)\left(1-\delta \right)\right]}{\left[1-\left(1-\gamma_{d}\right)\left(1-\gamma_{E}\right)\right]\left[1-\left(1-\gamma_{d}\right)\left(1-\delta \right)\right]}R_{1,2}\right)}^{-1}$$

So far, we have obtained all the results in Lemma 2. This completes the proof.

## References

[1] Kaul S., Gruteser M., Rai V., Kenney J. Minimizing age of information in vehicular networks. Proceedings of the 8th Annual IEEE Communications Society Conference on Sensor, Mesh and Ad Hoc Communications and Networks (SECOM).

[2] Kaul S., Yates R., Gruteser M. Real-time status: How often should one update?. Proceedings of the IEEE International Conference on Computer Communications (INFOCOM).

[3] Kaul S.K., Yates R.D., Gruteser M. Status updates through queues. Proceedings of the 46th Annual Conference on Information Sciences and Systems.

[4] Sun Y., Uysal-Biyikoglu E., Yates R., Koksal C.E., Shroff N.B. Update or wait: How to keep your data fresh. Proceedings of the 35th Annual IEEE International Conference on Computer Communications.

[5] Costa M., Codreanu M., Ephremides A. (2016). On the Age of Information in Status Update Systems with Packet Management. IEEE Trans. Inf. Theory.

[6] Kam C., Kompella S., Nguyen G.D., Wieselthier J.E., Ephremides A. (2018). On the Age of Information with Packet Deadlines. IEEE Trans. Inf. Theory.

[7] Inoue Y. Analysis of the Age of Information with Packet Deadline and Infinite Buffer Capacity. Proceedings of the IEEE International Symposium on Information Theory (ISIT).

[8] Inoue Y., Masuyama H., Takine T., Tanaka T. (2019). A General Formula for the Stationary Distribution of the Age of Information and Its Application to Single-Server Queues. IEEE Trans. Inf. Theory.

[9] Yates R.D., Kaul S.K. (2019). The Age of Information: Real-Time Status Updating by Multiple Sources. IEEE Trans. Inf. Theory.

[10] Pappas N., Gunnarsson J., Kratz L., Kountouris M., Angelakis V. Age of information of multiple sources with queue management. Proceedings of the IEEE International Conference on Communications (ICC).

[11] Kosta A., Pappas N., Ephremides A., Angelakis V. (2019). Age of information performance of multiaccess strategies with packet management. J. Commun. Netw..

[12] Dogan O., Akar N. (2021). The Multi-Source Probabilistically Preemptive M/PH/1/1 Queue with Packet Errors. IEEE Trans. Commun..

[13] Kam C., Kompella S., Nguyen G.D., Ephremides A. (2016). Effect of Message Transmission Path Diversity on Status Age. IEEE Trans. Inf. Theory.

[14] Javani A., Zorgui M., Wang Z. (2025). Age of Information for Multiple-Source Multiple-Server Networks. IEEE Trans. Netw..

[15] Chen Z., Yang T., Pappas N., Yang H.H., Tian Z., Wang M. (2025). Improving Information Freshness via Multi-Sensor Parallel Status Updating. IEEE Trans. Commun..

[16] Ramakanth R.V., Tripathi V., Modiano E. (2025). Monitoring Correlated Sources: AoI-Based Scheduling is Nearly Optimal. IEEE. Trans. Mob. Comput..

[17] Moradian M., Dadlani A., Khonsari A., Tabassum H. (2024). Age-Aware Dynamic Frame Slotted ALOHA for Machine-Type Communications. IEEE Trans. Commun..

[18] Tang Z., Yang N., Sadeghi P., Zhou X. (2023). Age of Information in Downlink Systems: Broadcast or Unicast Transmission?. IEEE J. Sel. Areas Commun..

[19] Wang Q., Chen H. (2023). Age of Information in Reservation Multi-Access Networks with Stochastic Arrivals: Analysis and Optimization. IEEE Trans. Commun..

[20] Fountoulakis E., Charalambous T., Ephremides A., Pappas N. (2023). Scheduling Policies for AoI Minimization with Timely Throughput Constraints. IEEE Trans. Commun..

[21] Zheng H., Xiong K., Fan P., Zhong Z., Letaief K.B. (2021). Age of Information-Based Wireless Powered Communication Networks with Selfish Charging Nodes. IEEE J. Sel. Areas Commun..

[22] Feng S., Yang J. (2022). Precoding and Scheduling for AoI Minimization in MIMO Broadcast Channels. IEEE Trans. Inf. Theory.

[23] Tripathi V., Talak R., Modiano E. (2019). Age of Information for Discrete Time Queues. arXiv.

[24] Kosta A., Pappas N., Ephremides A., Angelakis V. Non-linear Age of Information in a Discrete Time Queue: Stationary Distribution and Average Performance Analysis. Proceedings of the IEEE International Conference on Communications (ICC).

[25] Kosta A., Pappas N., Ephremides A., Angelakis V. (2021). The Age of Information in a Discrete Time Queue: Stationary Distribution and Non-Linear Age Mean Analysis. IEEE J. Sel. Areas Commun..

[26] Akar N., Dogan O. (2021). Discrete-Time Queueing Model of Age of Information with Multiple Information Sources. IEEE Internet Things J..

[27] Zhang J., Xu Y. On Age of Information for Discrete Time Status Updating System with Ber/G/1/1 Queues. Proceedings of the IEEE Information Theory Workshop (ITW).

[28] Zhang J., Xu Y. On Age of Information for Discrete Time Status Updating System with Infinite Size. Proceedings of the IEEE Information Theory Workshop (ITW).

[29] Zhang J., Xu Y. (2022). Age Analysis of Status Updating System with Probabilistic Packet Preemption. Entropy.

[30] Zhang J., Xu H., Cao D., Xu Y. (2024). Discrete Age of Information for Bufferless System with Multiple Prioritized Sources. IEEE Internet Things J..

[31] Yang Y., Zhang B., Guo D., Xiong Z., Niyato D., Han Z. (2024). Can We Realize Data Freshness Optimization for Privacy Preserving-Mobile Crowdsensing with Artificial Noise?. IEEE. Trans. Mob. Comput..

[32] Kim D., Yun S., Lee S., Lee J., Quek T.Q.S. (2024). Reinforcement Learning-Based Sensing Decision for Data Freshness in Blockchain-Empowered Wireless Networks. IEEE Wirel. Commun. Lett..

[33] Costa M., Sagduyu Y.E. Timely NextG Communications with Decoy Assistance against Deep Learning-based Jamming. Proceedings of the IEEE International Conference on Communications Workshops (ICC Workshops).

[34] Ma Y., Liu K., Liu Y., Zhu L. (2024). Timeliness and Secrecy-Aware Uplink Data Aggregation for Large-Scale UAV-IoT Networks. IEEE Internet Things J..

[35] Yang Y., Hanzo L. (2023). Permutation-Based Short-Packet Transmissions Improve Secure URLLCs in the Internet of Things. IEEE Internet Things J..

[36] Yang Y. Secure and Timely Status Updates in the IoT using Short-Packet Permutation-Based Transmissions. Proceedings of the IEEE 98th Vehicular Technology Conference (VTC2023-Fall).

[37] Wang Q., Chen H., Mohapatra P., Pappas N. Secure Status Updates under Eavesdropping: Age of Information-based Secrecy Metrics. Proceedings of the IEEE Conference on Computer Communications Workshops (INFOCOM WKSHPS).

[38] Wyner A.D. (1975). The wiretap channel. Bell Syst. Tech. J..

[39] Xu H., Hu Y., Zhu Y., Yuan X., Schmeink A. (2024). Achieving Secure and Fresh Information Updates via Short-Packet Communications. IEEE Wirel. Commun. Lett..

[40] Yuan F., Tang S., Liu D. (2024). AoI-Based Transmission Scheduling for Cyber Physical Systems Over Fading Channel Against Eavesdropping. IEEE Internet Things J..

[41] Zhang J., Xu H., Zheng A., Cao D., Xu Y., Lin C. (2025). Transmitting Status Updates on Infinite Capacity Systems with Eavesdropper: Freshness Advantage of Legitimate Receiver. Entropy.

